# The effect of temperature on phytoplankton physiology: a mesocosm and modeling study

**DOI:** 10.1128/spectrum.00457-25

**Published:** 2025-09-18

**Authors:** Gabrielle Armin, Gergely Boros, Mariann Kis, Máté Burányi, Hajnalka Horváth, Krisztina Krassován, Takako Masuda, Gábor Bernát, Keisuke Inomura

**Affiliations:** 1 Graduate School of Oceanography, University of Rhode Island, Narragansett, Rhode Island, USA; 2 Zooplankton and Ecological Interactions Research Group, HUN-REN Balaton Limnological Research Institute 272133 , Tihany, Hungary; 3 Aquatic Botany and Microbial Ecology Research Group, HUN-REN Balaton Limnological Research Institute 272133 , Tihany, Hungary; 4 Fisheries Resources Institute, Japan Fisheries Research and Education Agency, Shinhamacho 13519, Shiogama, Miyagi, Japan; Connecticut Agricultural Experiment Station, New Haven, Connecticut, USA

**Keywords:** phytoplankton, temperature, elemental stoichiometry, macromolecular allocation, model, mesocosm, climate change

## Abstract

Strategies that focus on reducing nutrient loading to freshwater lakes have historically been successful in improving water quality by curbing large phytoplankton blooms. However, as waters warm, little is known about the resultant phytoplankton physiology and ensuing perturbations in the food web that may occur. Here, we designed a mesocosm experiment to investigate the impact of warming water on phytoplankton physiology and further validate a previously developed, coarse-grained model that predicts the key aspects of phytoplankton physiology, including elemental stoichiometry and macromolecular allocation, across varying temperatures. We found that higher temperatures double the maximum cellular density (cells L^−1^) of phytoplankton, suggesting that high temperature stimulates cell division over maximizing carbon storage. Also, the cells in warmer waters dedicate fewer resources to proteins and RNA production, leading to higher fractions of carbon allocated to storage. This work illustrates the potential impact warming waters may have on the ecosystem, as higher fractions of carbohydrates are often associated with less nutritious food for higher trophic levels.

**IMPORTANCE:**

We take a novel approach to investigating the impact of warming on phytoplankton physiology by utilizing mesocosms and a coarse-grained cellular model. Previous work in this field tends to use idealized laboratory experiments, mesocosms, or models alone. By synthesizing model and mesocosm results, we test the model’s ability to capture physiology in semi-natural environments. We conducted this experiment under phosphorus limitation and saw high cell densities in the heated, treatment tanks. Thus, warming waters may negate some successful management practices that curb eutrophication. With increased temperatures, we also observed increased N:P values in both the experimental and model results, which may be due to the combined effects of a lack of P storage, fewer enzymes required, and a corresponding decrease in RNA production. Our model predictions closely aligned to mesocosm observations, suggesting the capability of our model to represent lower trophic organisms in ecosystem models.

## INTRODUCTION

Freshwater lakes are culturally and economically significant to the regions surrounding them. Besides recreation and ecotourism, lakes also provide several other ecosystem services, such as fresh drinking water, food production, nutrient cycling, and primary production (1–4). Due to their significance, these vulnerable systems are monitored and investigated globally (5–10) to identify key environmental drivers of large phytoplankton blooms, which can lead to decreased water quality. Phytoplankton are the assembly of autotrophic, unicellular organisms, which serve as primary producers at the base of aquatic food webs. Ubiquitous in aquatic environments, their role in nutrient transformation and cycling is essential to the fitness of aquatic ecosystems, including organisms at higher trophic levels within these environments (11, 12).

As with all photoautotrophic organisms, phytoplankton rely on light, nutrients, and a suitable environment for optimal growth; thus, environmental conditions strongly determine physiology and overall community structure (13, 14). Light, or irradiance, varies with depth and the concentration of biomass where irradiance decreases with increasing depth and increasing biomass due to a phenomenon where phytoplankton cells at the surface may shade cells below, which can lead to physiological adaptations (15). Regarding light, its irradiance changes continuously on slow and rapid time scales, and rates of photosynthesis vary, often dependent on the species (16). High irradiance could even inhibit growth and/or damage cells by photoinhibition (17, 18). Nutrient (i.e., nitrogen or phosphorus) limitation may also inhibit growth since phytoplankton strongly rely on these elements as essential components of macromolecules, such as proteins and RNA (12). In freshwater environments, phosphorus limitation is particularly important, as it is generally accepted that in these systems, globally, phosphorus is most often the limiting resource (19). On the cellular level, phosphorus limitation directly impacts growth and growth-related molecules, as phosphorus is a key element in RNA (20, 21). This has a larger effect in the ecosystem, as higher trophic organisms, such as zooplankton, require certain elemental ratios of nutrients, especially for phosphorus, from their food source to remain physiologically fit (22–24). Additionally, nutrient limitation may reorganize the community structure of phytoplankton due to the high surface area to volume ratios of smaller phytoplankton cells, such as cyanobacteria (25). The small cell size allows for more efficient nutrient acquisition (26–29), which may help these smaller cells outcompete larger eukaryotic phytoplankton. Other than light and nutrient levels, temperature is a key environmental condition that affects phytoplankton on both cellular and community levels. Temperature not only causes varied allocation to cellular processes, like biosynthesis or storage (30, 31), but can also cause cellular damage at high temperatures by denaturing proteins (32). Laboratory studies (33–39) have investigated changes in phytoplankton metabolic rates with temperature and frequently documented decreasing cell size and increased growth rates. Previous mesocosm studies (40–45) characterized the redistribution in community assemblages of phytoplankton and suggested a decrease in predictability of future annual cycles. Although there is a large effort to characterize phytoplankton response to increasing temperatures, there still remain outstanding questions and low predictability of resultant physiology.

Therefore, a phytoplankton mechanistic model may be useful to efficiently quantify and predict the relationship between the cellular response and the surrounding environment (e.g., temperature). Previously, we developed a temperature-dependent, mechanistic model, the Cell Flux Model of Phytoplankton (CFM-Phyto-T) (30), which predicts elemental stoichiometry and macromolecular allocation of nitrogen (N), phosphorus (P), and carbon (C) as a function of temperature. This coarse-grained model is based on a previously developed model that allocates nutrients to biomolecules within four major macromolecular pools, namely, biosynthetic, photosynthetic, essential, and storage pools (46). We set the starting parameters in CFM-Phyto-T so that they aligned with previously published experimental parameters in a laboratory experiment (47) and compared the resulting predictions to the observations. With the increasing temperature, the cellular N:P increased, as demonstrated by both the laboratory results and model predictions. CFM-Phyto-T further predicted a decreasing nutrient allocation to RNA and proteins and an increasing dedication to C storage with increasing temperature. Although we successfully validated our model results to experimental data under idealized laboratory conditions, that approach was with many simplifications and, thus, may not describe natural processes well, especially at the ecosystem level.

Mesocosms are unique tools, as they provide a stepping stone between idealized laboratory experiments and field observations. These types of studies allow for a certain level of control while exposing treatments to semi-natural conditions. In this study, we conducted a mesocosm experiment to investigate the impact of increasing temperature on phytoplankton physiology at the HUN-REN Balaton Limnological Research Institute in Tihany, Hungary. We paired the observations with model predictions of CFM-Phyto-T to ensure our model predictions are equally accurate in both field-like (semi-natural) and laboratory conditions. Our guiding questions are as follows: (i) How does phytoplankton physiology, characterized by elemental stoichiometry and macromolecular allocation, vary with increasing temperature? (ii) Can CFM-Phyto-T be applied to model biological processes occurring in mesocosm systems? Quantifying changes in phytoplankton cellular physiology is essential to understanding the macromolecular allocation and the nutritional quality these primary producers provide in the environment. Eventually, these findings may illustrate that CFM-Phyto-T remains a realistic representation of phytoplankton and could be used to effectively connect the environment and biology in larger ecosystem models.

## MATERIALS AND METHODS

### Mesocosm site

The outdoor mesocosm system at the HUN-REN Balaton Limnological Research Institute in Tihany, Hungary is situated approximately 20 m from Lake Balaton, the largest freshwater lake in central Europe located in western Hungary (Fig. S1). This mesocosm system houses 12 plastic tanks with a volume of 5 m³ and a water depth of 1.5 m. Each tank has a net-like covering that allows light to pass through while capturing large debris, multiple *in-situ* sensors that record measurements as often as every 10 min, and electric heating wires controlled by a programmable logic controller (PLC). For a list of the physiochemical parameters recorded, see Table S1 in the Supporting information. The mesocosms are contained within a fence and protected by wires overhead to dissuade any wildlife (i.e., birds) from entering the area.

### Experimental set-up and sampling method

**Fig 1:**
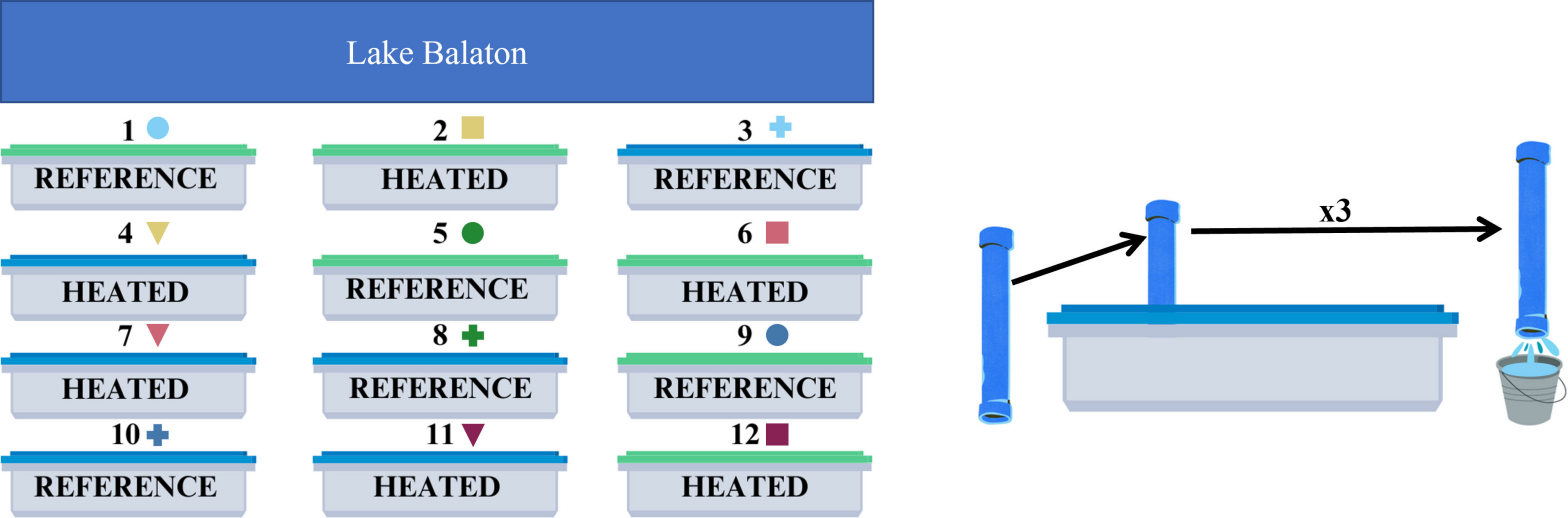
The outdoor mesocosm system at the Balaton Limnological Research Institute is situated on the shores of Lake Balaton (blue rectangle). We inoculated tanks (labeled by numbers above) with monocultures of a eukaryotic species of phytoplankton, *Raphidocelis subcapitata* (green top), and a prokaryotic strain, *Cyanobium gracile* (blue top). We heated half of the tanks (heated) at 4.5°C above the ambient temperature, while the other half (reference) remained at ambient temperature. Symbols next to the tank number correspond to the markers used throughout the manuscript to represent each tank. In order to sample from the mesocosms, we used a 4-m PVC tube to take water column samples in triplicate and emptied each replicate into one bucket corresponding to the tank number. From this bucket, we took samples for laboratory analysis.

For our experiment, half of the tanks were heated at 4.5°C above the ambient lake water temperature, while the other half remained at the ambient, here referred to as reference, temperature. We chose this heating regime based on the most pessimistic scenario (Representative Concentration Pathway 4.5 [RCP 4.5]) determined by the Intergovernmental Panel on Climate Change (IPCC) (48). We filled each tank with water from Lake Balaton that passed through a gravel, sand, and UV filter to ensure our monocultures were the only phytoplankton present in the mesocosms. On Day 0, we inoculated half of the tanks with a eukaryotic strain of phytoplankton, *Raphidocelis subcapitata* (Korshikov) (formerly known as *Selenastrum capricornutum*) (49) Algal Culture Tihany (ACT) 97101, and the other half with a prokaryotic strain, *Cyanobium gracile*, ACT 9802, thereafter referred to as *Raphidocelis* and *Cyanobium*. We chose these two different phytoplankton species to explore the differences between eukaryotic and prokaryotic cells. Therefore, of the six eukaryotic tanks, three were heated, and three were kept at the reference temperature. Likewise, three of the prokaryotic tanks were heated, while the other three were left at the reference temperature (Fig. 1A). Additionally, on Day 0, we added nutrients to the tanks to create a phosphorus-limited environment (55 g NaNO_3_, 1.811 g KH_2_PO_4_; 100 mol N:1 mol P), as the filtered water from the oligotrophic lake may not have sparked detectable and measurable blooms. This was the only nutrient addition chosen to align with freshwater systems more generally, as many are considered to be most limited by phosphorus (19).

Each tank had a designated 2 m-long PVC tube and a bucket that we used for sampling the mesocosms (Fig. 1B). To obtain well-mixed and representative samples from the mesocosm, we used the PVC tube to retrieve water column grab samples from the surface to bottom of the mesocosm. We took three samples from around the tank and added each water column grab to the same designated bucket. From this bucket, we took necessary samples for further analysis. We sampled the mesocosm in this manner each day to measure cell suspension density and approximately every third day to measure and collect samples for cellular stoichiometry, water column nutrient analysis, and macromolecular composition (e.g., chlorophyll, RNA, protein, etc.). The *in-situ* sensors were used to measure temperature, dissolved oxygen, pH, conductivity, and light irradiance. For an extensive list of the parameters, the sampling rate, and corresponding quantification method, see Table S1.

### Sample preparation and analysis

To determine cell number and growth, we collected 10 mL of well-mixed samples from each tank (i.e., from their respective bucket). We pipetted 1 mL aliquot into a cylindrical cuvette and placed it on the PARTEC CyFlow Space flow cytometer (Partec GmbH, Görlitz, Germany) capillary. For each tank, we measured the cell suspension density in triplicate. We used the instrumental FloMax software to calculate the total number of analyzed particles in the range of 0.1 to 50 µm and the concentration per mL. To analyze subpopulations within the samples in terms of cell size, shape, and granularity, we applied the gating analysis within the software. During the cytometric analysis, pigment autofluorescence (excitation: 488 nm, detection: 530–680 nm) was also recorded to validate the selection of cells within the total number of measured particles. To calculate the specific growth rate from the cell suspension density, we used equation 1:


(1) $$\mu =\frac{ln\left(C_{f}-C_{i}\right)}{dt}$$

where μ is the growth rate (d^−1^), and C_i_ and C_f_ are the initial and final cell counts (cells mL^−1^), respectively.

To determine nutrient levels in the biomass and water column, we both analyzed unfiltered samples from each respective bucket and filtrates obtained by passing water samples (see Table S2 for volumes of samples) through glass fiber filters (Whatman GF/F). To estimate biomass, we weighed the filters both before passing the samples over and after they were dried at 60°C in a drying oven. We determined the nitrate and nitrite concentrations in the filtrates by using the Cd-reduction method of Elliott and Porter (50, 51). The concentrations of total nitrogen (TN) and total dissolved nitrogen (TDN) in the unfiltered water samples and corresponding filtrates were determined as soluble nitrate after a digestion process with sodium hydroxide (NaOH) and potassium peroxide-disulfate (K_2_S_2_O_8_) (52). We determined the concentration of soluble reactive phosphorus (SRP), available for phytoplankton, from the filtrates by a modified method, as in Murphy and Riley (51, 53). The concentrations of total phosphorus (TP) and total dissolved phosphorus (TDP) were measured from unfiltered water samples, and corresponding filtrates were measured similarly after an hour of autoclaving in the presence of K_2_S_2_O_8_ (54). For particulate phosphorus (PP), we placed the filter in a test tube with 10 mL MQ water, digested the sample in the autoclave, and determined its concentration with the same above method. We used a SPECORD PLUS UV/VIS double-beam spectrophotometer (Endress + Hauser Analytik Jena, Jena, Germany) to measure the nutrient concentrations in triplicate with a coefficient of variation less than 5%. We determined the particulate carbon (PC) and nitrogen (PN) contents of the dried samples by an automated elemental analyzer interfaced to an Integra-2 isotope ratio mass spectrometer (Sercon Limited, Crewe, UK) (55). We used a variety of methods to quantify different macromolecules within cells throughout the experiment (Table S1). To determine chlorophyll*-a* concentrations, we filtered our samples (volumes ranging from 50 to 300 mL, see Table S3) onto the GF/F filters, placed the filters into test tubes, and added 5 mL of a methanol solution. We placed the tubes into a hot water bath and removed the samples 1 min after reaching their boiling point (64.7°C). We then measured the chlorophyll-*a* concentration using a SPECORD PLUS spectrophotometer (56).

### Model simulations

**Fig 2:**
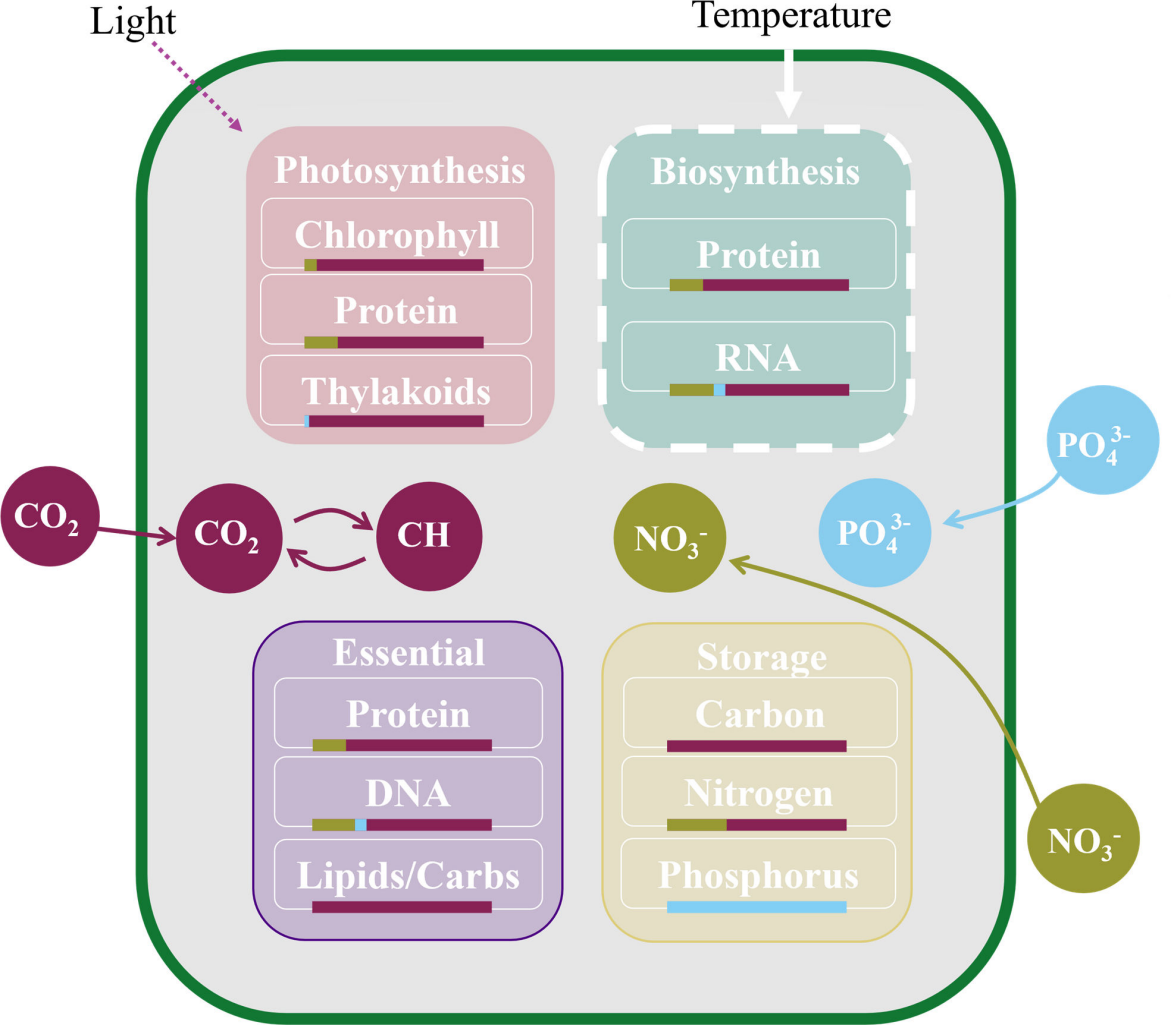
The temperature-dependent Cell Flux Model of Phytoplankton (CFM-Phyto-T) allocates phosphorus (blue circles), nitrogen (green circles), and carbon (maroon circles) to basic cellular functions (rounded squares). We designate these cellular functions into four pools: photosynthetic molecules, biosynthetic molecules, essential molecules for cell survival and structure (labeled as “Essential”), and nutrient storage. The white dashed lines highlight that the biosynthetic pool is affected by temperature effects in the model. Bars underneath each biomolecule represent an average fraction of carbon, nitrogen, or phosphorus within the molecule (46). Model schematic adapted from Armin and Inomura (2021) (30).

At the conclusion of the experiment, we compared our experimental data to model predictions using the temperature-dependent Cell Flux Model of Phytoplankton (CFM-Phyto-T). CFM-Phyto-T is a coarse-grained model (Fig. 2) that we used previously to predict elemental stoichiometry and macromolecular allocation of carbon, nitrogen, and phosphorus with changing temperature and compared to laboratory results (30, 47). The model predicts allocation to major pools of functional biomolecules, including photosynthetic, biosynthetic, storage, and essential biomolecules necessary for basic cellular structure and survival. Nutrients are allocated to these biomolecules, as they comprise the highest fractions of total mass of the given element (N, P, or C) within the cell (21, 46, 57, 58). Contribution from dissolved small molecules, including ATP, is relatively small in mass (59) and not explicitly represented in model allocation equations but implicitly included in the pool of essential biomolecules. Here, we employ CFM-Phyto-T to examine how closely the model predictions compare to experimental data obtained under semi-natural conditions. In our model simulations, we set the initial nutrient regime to be phosphorus-limited for all tanks and used the growth rate, light intensity, temperature, and nutrient concentrations from the *in-situ* measurements to constrain the model predictions of elemental stoichiometry and macromolecular allocation. We used the *in-situ* data obtained between 7:00 AM and 9:00 AM to align with the time of our grab sampling, which we used for determining elemental stoichiometry and macromolecular concentrations. Additionally, we tuned cellular parameters, such as the maintenance cellular respiration rate, to align with *Raphidocelis* for the eukaryotic model simulation. For the prokaryotic model simulation, we kept the original CFM-Phyto parameters, which were used previously (60), to represent the average elemental stoichiometry and macromolecular allocation within a phytoplankton community. We determined significant differences between parameters by performing two-factor analysis of variance (ANOVA) tests and using linear regression to compare model predictions with experimental data.

## RESULTS

Throughout the duration of the experiment, the mesocosm tanks inoculated with the prokaryotic cells, *Cyanobium gracile*, became contaminated with other species (Fig. S2). The dominating contaminant species was *Scenedesmus acutus*, a colony-forming, freshwater green algae. However, there were other species present in the tanks; thus, the results presented for these tanks are no longer considered a monoculture and rather act as a community of phytoplankton, termed “Mixed Population,” thereafter.

### Mesocosm observations

**Fig 3:**
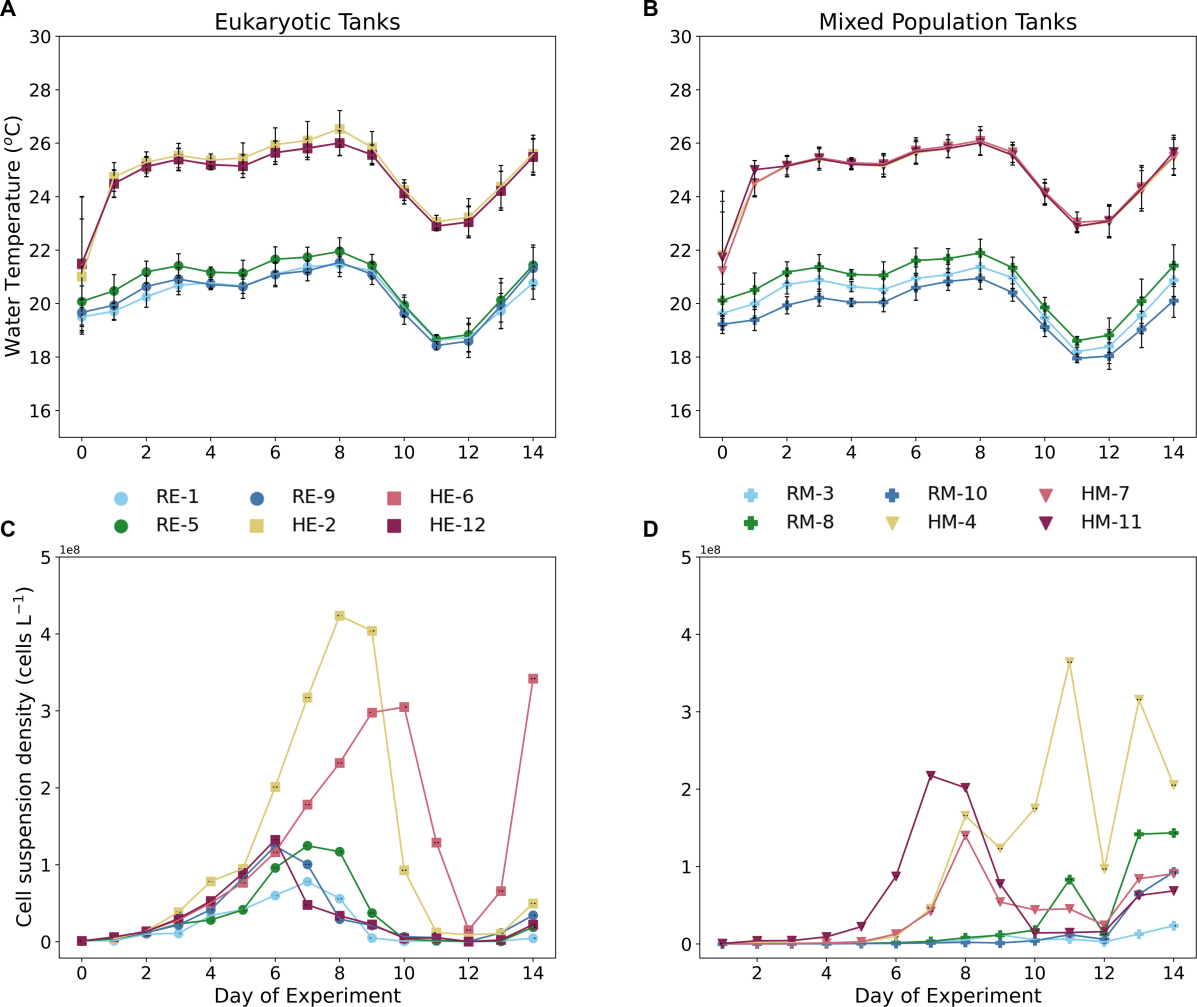
Water temperature (°C) in eukaryotic (**A**) and mixed-population tanks (**B**) and average cell suspension densities (cells L^−1^) in eukaryotic tanks (**C**) and mixed-population tanks (**D**) for the duration of the mesocosm experiment. Heated tanks are represented by warm-colored squares (eukaryotic) and triangles (mixed population), while reference tanks are represented by cool-colored circles (eukaryotic) and plus-signs (mixed population). Error bars signify the standard deviation among the replicates. In the legend, the first letter in the label refers to treatment type (i.e., reference, R, or heated, H), while the second denotes the species type in the tank (eukaryotic, E, or mixed population, M) and the number of the specific tank.

Over the course of the 14-day experiment, the temperature ranged (Fig. 3A and B) from 18.6 to 21.5°C, with the lowest and highest temperature days occurring on days 11 and 8, respectively. We started the heat treatment in the respective tanks on Day 0; thus, the desired difference between the two temperature regimes was not achieved until Day 1. Throughout the experiment, there was minimal variation in temperature between replicates of each treatment. The variation in the dissolved oxygen (DO) concentration in the water column (Fig. S2) among the tanks became more distinct through the duration of the experiment, with Day 12 having the most variation (77%) among eukaryotic tanks and Day 13 (73%) in the mixed population tanks. In both the eukaryotic and mixed-population tanks, the heated tanks peaked in DO concentration before the reference tanks. The light sensor that measured the irradiance of the tanks was located at the top of each tank facing the interior; thus, the irradiance was calculated from the reflectance off the water. Tanks 11 and 12 had consistently lower average irradiance values (Fig. S3) and had the highest shade coverage throughout the day. Day 10 had the lowest, while Day 0 had the highest irradiance levels across all tanks. There was no significant difference between the measured irradiances between treatments in the eukaryotic tanks throughout the experiment (Fig. S3). However, the measured irradiances between the heated and reference mixed-population tanks were statistically significant on days 7 and 8 (P* < 0.05) (Fig. S3).

The total dissolved nitrogen (TDN) in the water column (Fig. S2) steadily decreased after Day 4 through the duration of the experiment in all tanks. The depletion of TDN was most severe in the heated mixed-population tanks having a reduction of about 50% throughout, whereas the eukaryotic and reference mixed population tanks had 18.5 and 24% reductions. There was a little variation in TDN among treatments throughout the experiment in the eukaryotic tanks. Conversely, the pattern of total dissolved phosphorus (TDP) in the water column (Fig. S2) varied among the tanks. In the heated eukaryotic tanks, the maximum concentrations occurred on Day 0 for Tank 12, then steadily decreased through the duration of the experiment. In tanks 2 and 6, the concentration steadily decreased to a local minimum on Day 7, increased on Day 11, and then dropped again on the last day of the experiment. The TDP concentration in the reference eukaryotic tanks followed a similar pattern, decreasing steadily to a local minimum on Day 7, jumping in concentration on Day 11, and falling again on Day 14. The mixed-population tanks had more variation between heated and reference tanks in the first week of the experiment. However, the tanks had similar trends of starting at a maximum concentration steadily declining. Between days 4 and 7, the concentration of TDP in the heated mixed-population tanks dropped, whereas it dropped in the reference tanks between days 7 and 11.

**Fig 4:**
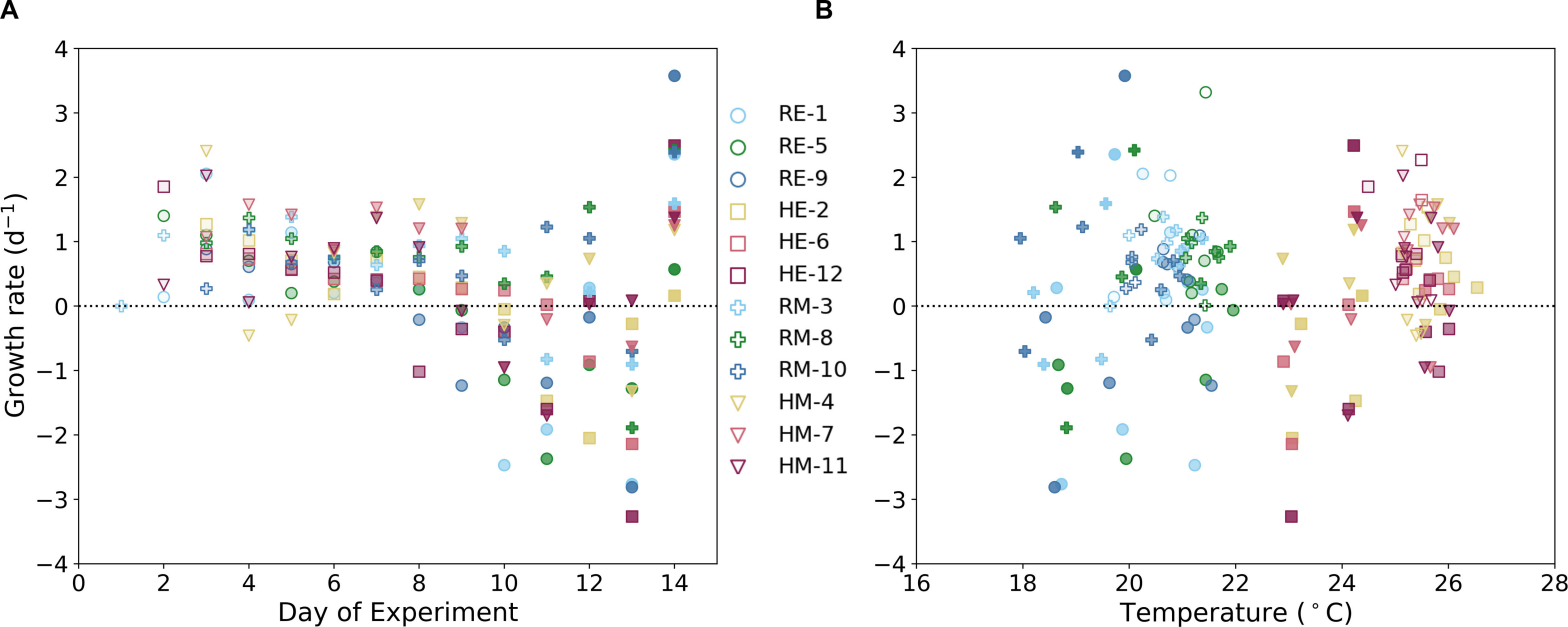
Growth rates (d^−1^) in each tank across each day of the experiment (**A**) and with increasing temperature (**B**). Points are shaded by the day in the experiment; as the day in experiment increases, the shading darkens.

In both the eukaryotic monoculture and the mixed-population tanks, heated tanks had higher maximum cell densities compared to the reference tanks (Fig. 3C and D). In the eukaryotic tanks (Fig. 3C), tanks 5 (reference) and 2 (heated) had the highest levels of cell suspension density on days 7 and 8. The other reference eukaryotic tanks, tanks 1 and 9, peaked in cell abundance on days 7 and 6, while the remaining heated tanks, tanks 6 and 12, peaked on days 8 and 10. Each heated tank had higher maximum cellular densities compared to the reference, with Tank 2 having the highest maximum with 4.24 × 10^8^ ± 0.06 × 10^8^ cells L^−1^ (%SD = 1.3), while the highest maximum in the reference tanks occurred in Tank 5 with 1.25 × 10^8^ ± 0.06 × 10^8^ cells L^−1^ (%SD = 4.5). Cell densities in heated tanks 2 and 6 were significantly different than the remaining tanks. Day 14 had an uptick in cell abundance in all eukaryotic tanks, which led us to terminate the experiment following this observation. Among the mixed-population tanks, the highest cell suspension density occurred in Tank 4 with 3.64 × 10^8^ ± 0.71 × 10^8^ cells L^−1^ on Day 11 (%SD = 19.3). The other heated tanks reached maximum cell densities earlier on days 7 and 8. The reference mixed-population tanks all peaked on the last day of the experiment, Day 14, with Tank 8 having the highest abundance with 1.44 × 10^8^ ± 0.10 × 10^8^ cells L^−1^ (%SD = 6.86). The distribution of growth rates (d^−1^) across the duration of the experiment (Fig. 4A) largely explains the cell suspension density distribution. Toward the beginning of the experiment, all tanks, except HM-4, showed positive growth until Day 8 of the experiment. Afterwards, growth rates became increasingly negative until the last day of the experiment, where we saw a corresponding uptick in cell suspension density. There was no significant difference (Fig. 4B) between the measured growth rates between heated and reference tanks (P* > 0.05). However, the standard deviation among the heated tanks’ measured growth rate was lower than the standard deviation for the reference tanks’ growth rates.

### Comparing model predictions with observations

**Fig 5:**
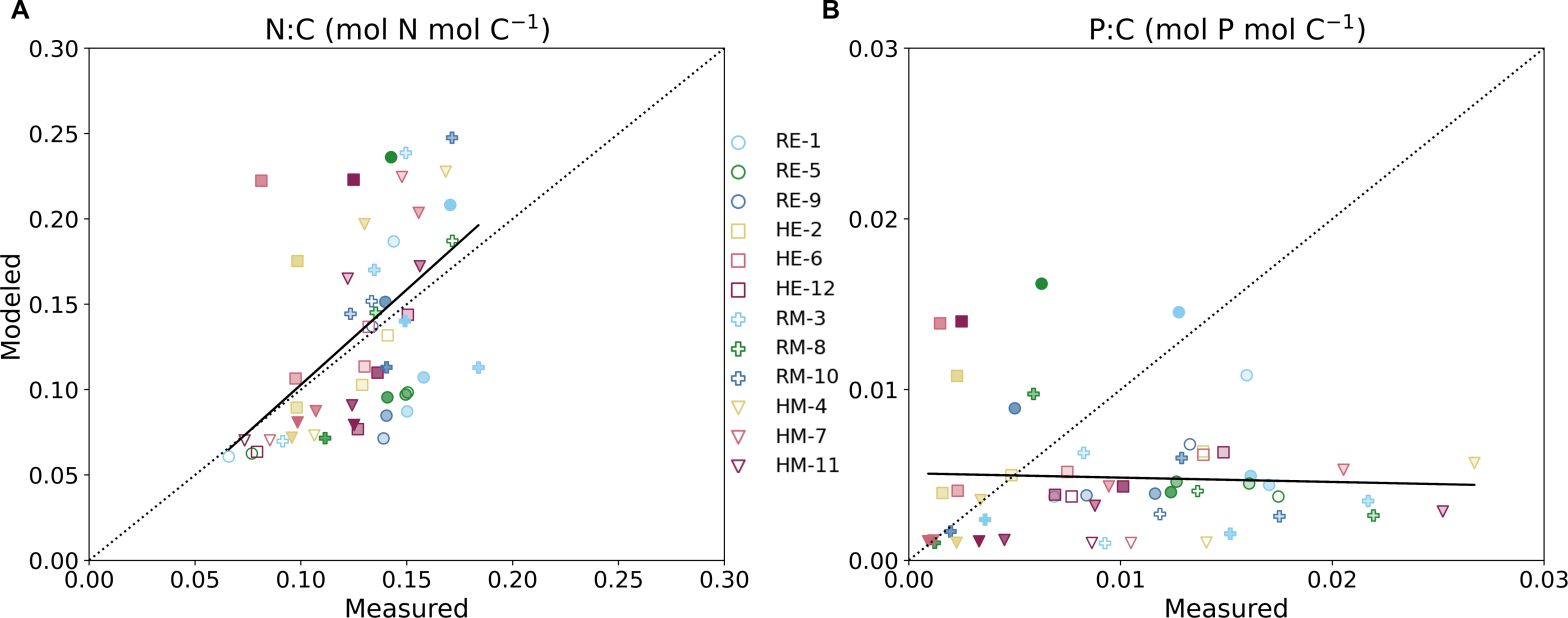
Modeled vs. measured values of N:C (mol N mol C^−1^) (**A**) and P:C (mol *P* mol C^−1^) (**B**) for all tanks. The fill or shading of each point represents the day of the experiment, with no fill being the first measurement on Day 4 and the opaque points those on Day 14. The dotted black line on both plots is a 1:1 line, while the solid line is the linear regression of the scatter. However, the *R*^2^ for N:C and P:C was 0.26 and 0.002, respectively, indicating low to no correlation.

Besides *in situ* parameters and cell abundance, we measured various intracellular parameters during the experiment and compared the results to modeled predictions of elemental stoichiometry and macromolecular allocation. Across all eukaryotic tanks and treatments, the nitrogen to carbon intracellular ratios (N:C) increased from day 0 to 4 (Fig. S5A and D). Then, the values plateaued in the reference tanks and slowly declined in the treated ones. Thus, the reference tanks had higher N:C values in both cases by the end of the experiment compared to the heated tanks. In the eukaryotic reference tanks, phosphorus to carbon ratios (P:C) steadily decreased (Fig. S5B). The heated eukaryotic tanks also demonstrated a decreasing tendency starting after Day 4 and generally had lower P:C ratios compared to the reference tanks. In the reference mixed-population tanks, P:C increased to a maximum on Day 7 and decreased steadily through the end of the experiment (Fig. S5E). The heated mixed-population tanks followed a similar trend, reaching a slightly lower maximum value on Day 4, and then generally decreased. For both treatments in all tanks, the N:P values (Fig. S5C and F) generally increased throughout the duration of the experiment. Heated tanks in both eukaryotic and mixed-population tanks frequently had higher values of N:P after Day 6 of the experiment. When we compared the measured N:C and P:C values in all tanks to model predictions (Fig. 5), the model predictions and observations for N:C were more closely related than for P:C. However, in each case, the low *R*^2^ values, 0.26 and 0.002 for N:C and P:C, respectively, indicate minimal correlation. The predicted N:C values for the earlier days of the experiment are situated closer to the line, and as the days progressed, the residuals increased for most of the tanks. Here, there are several instances when the model overestimates the value of N:C in the heated tanks compared to the reference tanks. Conversely, the model underestimated values of P:C early on in the experiment and became closer to observation as the days progressed.

**Fig 6:**
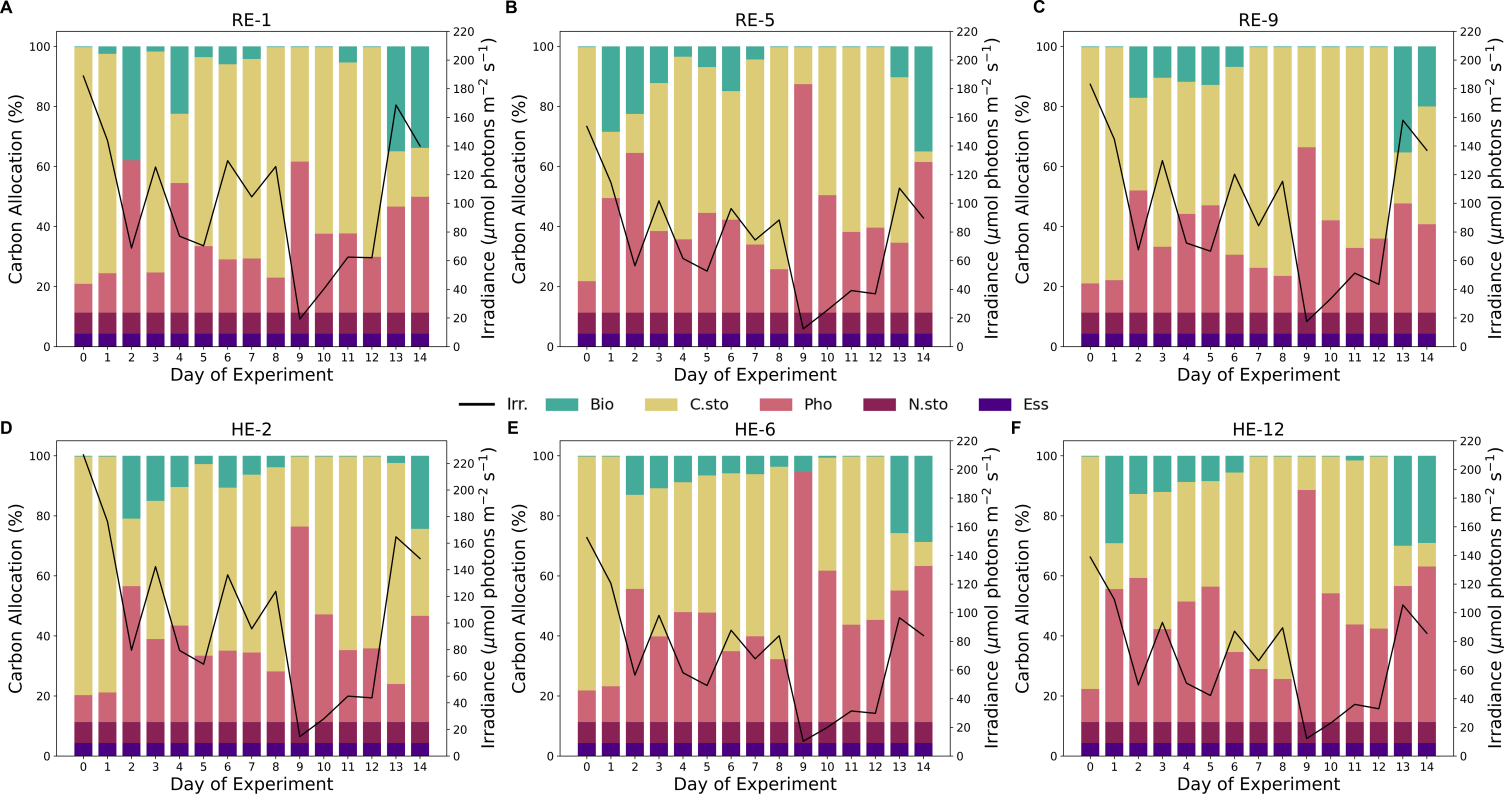
Modeled carbon allocation (%) to biosynthetic (teal), photosynthetic (pink), carbon storage (yellow), nitrogen storage (maroon), and essential (purple) macromolecules for each day of the experiment in the eukaryotic reference (**A-C**) and heated (**D-F**) tanks. Solid black lines represent the average irradiance (μmol photons m^−2^ s^−1^) at the time of sampling. Temperature, irradiance, nutrient concentrations, and growth rates measured in the mesocosms were used to constrain model predictions of allocation and elemental stoichiometry.

**Fig 7:**
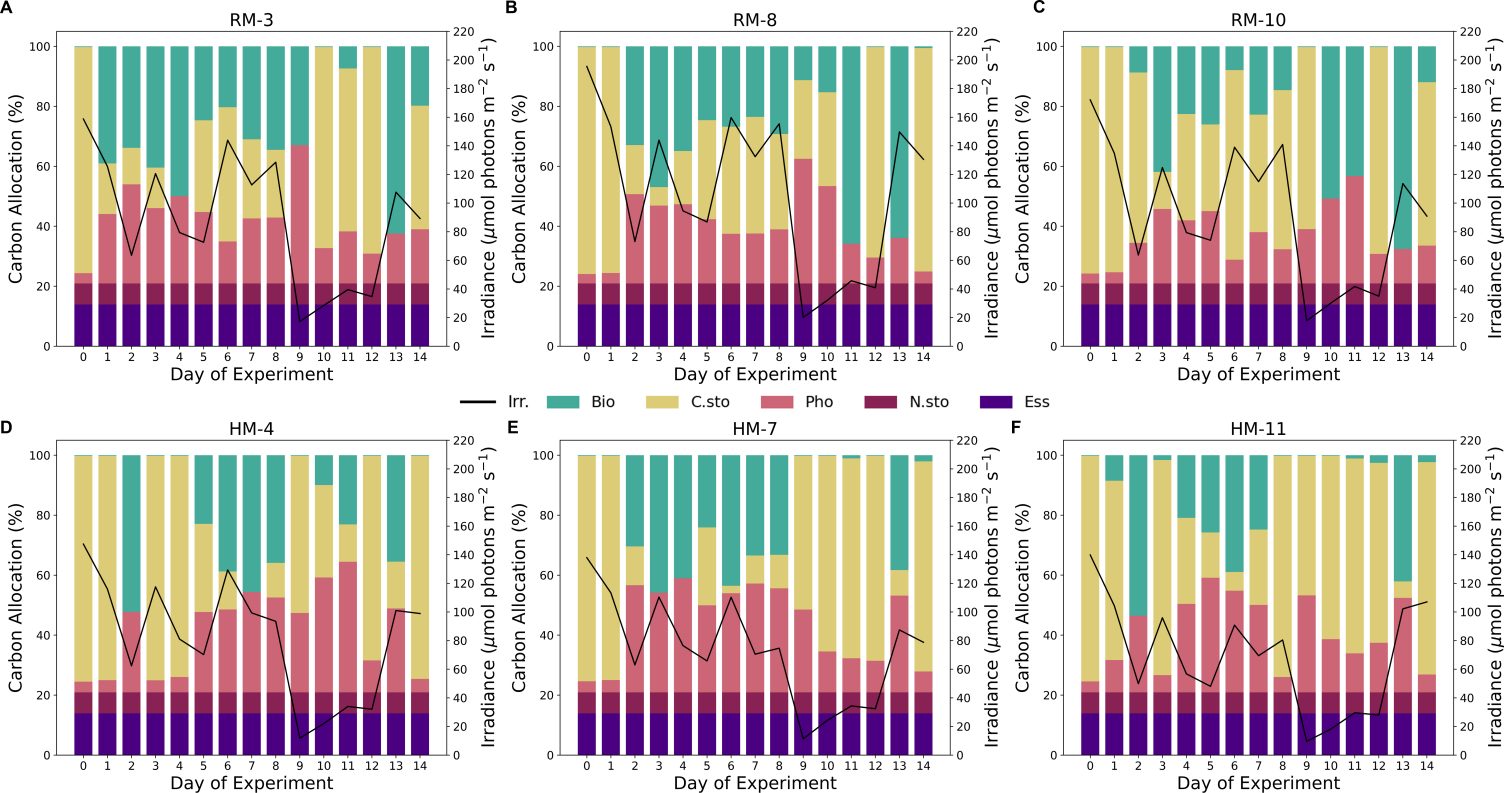
Modeled carbon allocation (%) to biosynthetic (teal), photosynthetic (pink), carbon storage (yellow), nitrogen storage (maroon), and essential (purple) macromolecules for each day of the experiment in the mixed-population reference (**A-C**) and heated (**D-F**) tanks. Solid black lines represent the average irradiance (μmol photons m^−2^ s^−1^) at the time of sampling. Temperature, irradiance, nutrient concentrations, and growth rates measured in the mesocosms were used to constrain model predictions of allocation and elemental stoichiometry.

Using CFM-Phyto-T, we predicted the macromolecular allocation of C, N, and P to various macromolecular pools within the cell. In the eukaryotic simulations, we used parameterized cellular constants that aligned with previous observations (46). However, for the tanks with mixed populations, we used the original parameters of the CFM-Phyto, as those parameters were shown to capture an average community well. In each tank, modeled C allocation (Fig. 6 and 7) to the photosynthetic, biosynthetic, and C storage macromolecules varied over the course of the experiment, while the dedication to essential and N storage macromolecules remained constant. Early in the experiment (days 0–8), model predictions in the eukaryotic tanks (Fig. 6) frequently showed higher dedication to biosynthetic macromolecules in the reference tanks. Biosynthetic machinery comprised a higher fraction in all tanks on days 13 and 14 likely due to increasing growth rates observed in tanks. In the mixed-population tanks, the biosynthetic fractions of C were larger than those in the eukaryotic tanks due to the higher parameterized, cellular constants in this simulation (Fig. 7).

The reference mixed-population tanks had the highest cell densities on the last day of the experiment (Fig. 3D), which coincided with the consistently high predicted C allocation to biosynthetic machinery on Day 13. Similarly, the heated tanks saw elevated dedication to biosynthetic machinery earlier in the experiment, near the day when their maximum cellular densities peaked. Day 9 had the lowest irradiance values during the experiment, which corresponded with the highest modeled fraction of C dedicated to photosynthetic machinery. The modeled fractions of C allocated to photosynthetic machinery were consistently higher in the eukaryotic tanks compared to the mixed-population tanks. However, in both cases, they followed a similar trend over the course of the experiment, which was inversely related to light irradiance (Fig. S6). Expectedly, C storage is inversely related to photosynthetic and biosynthetic macromolecules, the largest variations occurred on days 2 and 9 in the eukaryotic tanks (Fig. S6). Unlike the eukaryotic tanks, the mixed population tanks had high variation of predicted C allocation among the heated and reference tanks most days.

Similar to C allocation, the model predicted constant levels of N (Fig. S6 and S7) dedicated to essential and N storage macromolecules. Likewise, phytoplankton in the eukaryotic tanks (Fig. S7) allocated more N to photosynthetic machinery, while those in mixed-population tanks (Fig. S8) allocated a higher proportion of N to biosynthetic macromolecules. In most eukaryotic tanks, the last days of the experiment showed higher dedication to biosynthetic macromolecules, and heated tanks generally comprised smaller fractions than the reference tanks. Day 8 was a low in predicted N:C across eukaryotic tanks, with minimal N dedicated to biosynthetic macromolecules. The mixed-population tanks (Fig. S8) had a similar pattern emerge on Day 12, where the N:C was lower than that in previous days, and negligible N was dedicated to biosynthesis. As seen in C allocation, Day 9 had the highest fraction of N allocated to photosynthesis across eukaryotic tanks. However, this modeled fraction in the heated mixed-population tanks on the same day did not vary significantly from the other days. Contrary to N allocation, the model did not resolve any P to storage since we assumed P to be a limiting nutrient in our simulations (Fig. S8 and S9), reflecting our experimental conditions. The model predicted constant fractions of P allocated to essential macromolecules in both the eukaryotic and mixed-population tanks, which was significantly lower in the mixed-opulation tanks due to lower cellular constants within the model. P allocation is dominated by biosynthetic machinery, especially to RNA molecules, with a small proportion of P dedicated to thylakoid membranes, key to photosynthetic reactions in both prokaryotic and eukaryotic phytoplankton. All but one eukaryotic tank (Tank 6) (Fig. S9) had lower predicted values of P:C in days 8 through 12, forming a U-shaped pattern in modeled P:C immediately following high cellular densities that occurred before this period of the experiment. Reference mixed-population tanks (Fig. S10) peaked in predicted P:C later in the experiment compared to heated tanks. Additionally, these heated tanks had lower maximum P:C values and corresponding allocation to biosynthetic machinery compared to those of the reference tanks.

To understand the differences between modeled and observed data, we determined the concentration of chlorophyll throughout the experiment and compared these values with CFM-Phyto-T allocation predictions (Fig. S11). Compared to chlorophyll measurements, the model overestimated chlorophyll content in the early days of the experiment (days 0–4) but represented the heated and reference tanks with similar variation. Here, the model predictions of chlorophyll content and measurements were comparable, the ranges being within the same orders of magnitude. However, it seems the model predictions were somewhat underestimating the chlorophyll content, which can be explained by the self-shading effect in the mesocosm, which the model does not account for. Self-shading may lower the in-situ light intensity, inducing cellular acclimation toward a higher allocation of light-harvesting apparatus (61).

## DISCUSSION

### Cellular response

Throughout our experiment, we observed dense populations in the heated tanks and a contaminant species introduced toward the end of the experimental period. Emerging patterns in our data reveal why these events may have occurred and how this affected cellular physiology within the mesocosms. One major event that contributed to some of these patterns was frequent rain during the experiment. Starting the evening of the ninth day, heavy rains and cooler temperatures dominated and occurred through Day 11 of the experiment, which explains the observed gradual decline in temperature and minimum irradiance values across all tanks. Consequently, this period had consistently high relative C allocation to photosynthetic machinery to capture the low light that was available during this period to provide energy to the cells. This also emphasizes that the cells did not have enough energy for biosynthetic reactions and thus dedicated fewer nutrients to the biosynthetic macromolecular pool, prioritizing photosynthetic machinery. Moreover, plastids (organelles associated with photosynthesis) tend to have high N:P values due to the concentration of RuBisCo, a key enzyme involved in carbon fixation, and comparably small concentrations of P in thylakoid membranes (62), which is consistent with our observations of increased N:P values and allocation to photosynthetic machinery on Day 11. Although the tanks’ mesh covers kept large debris out, these heavy winds and rain may have attributed to the introduction of multiple new species in the mixed-population tanks via aeolian transport and, thereafter, the overall increase in cell densities. In the mixed-population tanks, we observed the rise of these new species shortly after the storm, whereas we did not see evidence of new species in the eukaryotic tanks until the final day of the experiment. As the population of *Raphidocelis* began to die out, the suspected main contaminant species, *Scendesmus acutus*, started to bloom, which prompted our decision to end the experiment after 14 days.

In both eukaryotic and mixed-population tanks, we observed the highest maximum cell densities in the heated tanks. Phytoplankton metabolism is certainly influenced by temperature (63). Warmer waters may lower activation energy requirements for biochemical reactions in cells and thus increase the cell division rates and overall cell suspension density of a phytoplankton bloom (64). However, there is a point past the optimal temperature, which may harm a cell, resulting in denatured proteins (65) and increased mortality (31). With a lower activation energy, the biochemical rates, such as those associated with biosynthesis, may increase; thus, the need for enzymatic catalysts decreases. For this reason, the predicted C allocation fractions of biosynthetic macromolecules are often larger in the reference tanks compared to the heated tanks. Similarly, we observed many instances in which the N:P values were higher in the heated tanks, corresponding with the results in previous studies (30). The N:P values increase under increased temperature in a P-limited environment due to the combined effects of a lack of P storage, fewer enzymes required, and a corresponding decrease in RNA production (30, 66). Furthermore, the eukaryotic reference tanks peaked in cell suspension density earlier than the heated tanks, although the population is comparable among reference and heated tanks on the days in which the reference tanks reach their maximum. Likely, the higher temperatures allow the population in the heated tanks to continue to grow, as available resources can be directed to C storage and reserved for new cells, whereas in the cooler tanks, the same resources are required to produce enzymes. Conversely, the mixed-population reference tanks peaked in cell suspension density on the last day after the heated tanks. These distributions of cell suspension density could be largely explained by the distribution of growth rates for the duration of the experiment. As opposed to the eukaryotic tanks, the prevailing population in this tank did not comprise species, which we inoculated. Therefore, the warmer waters may have aided the dominant, colonizing species to reach its maximum population density sooner. In the reference tanks, the introduced species took a similar amount of time to reach its maximum as *Raphidocelis* in the eukaryotic tanks. Additionally, the days in which we measured high growth rates and RNA concentrations were accurately depicted by the model, with these days having increased dedication to biosynthetic macromolecules. Lastly, the measured irradiance in the reference tanks was consistently higher than that of the heated tanks. As these sensors measure the reflectance from the water, this was most likely due to self-shading, absorption, and therefore less reflected light back on the sensors due to higher cell densities in the heated tanks. This is reflected in model predictions, as nutrient allocation to photosynthetic machinery was higher in heated tanks compared to the reference tanks.

In the eukaryotic tanks, we consistently predicted more resources allocated to photosynthetic machinery primarily due to our parameterization of the model. We increased a constant in CFM-Phyto-T that represents the maintenance carbohydrate consumption rate. This assumption increases the predicted fraction of photosynthetic machinery, which may align with the chloroplast packing previously seen in *Raphidocelis*. These cells are known for their elongated, crescent-shaped cells, which aid in light harvesting and in reducing the shading effect (67–69). We kept the original parameterization for the mixed-population tanks, as the original cell parameters are meant to represent an average community of phytoplankton.

### Colonizing species

Although cyanobacteria are expected to have advantages in warmer waters due to their small size (70–72) and higher temperature growth optima (73), our strain never replicated enough to signal that a bloom occurred. Instead, *S. acutus* was the predominant primary producer in the mixed-population mesocosms. This species has slightly smaller cells (74) (12–19 μm long, 3–7 μm wide) compared to *Raphidocelis (*75) (16–38 μm long, 5–8 μm wide) and can form four- to eight-cell colonies. This behavior aids the cells in evading grazers (76) but limits their nutrient uptake as the surface area to volume ratio decreases (77, 78). Decreased nutrient uptake may explain why we observed lower N:P values among the mixed-population tanks. Although within the mixed-population tanks, we observed higher N:P and lower concentrations of nutrients in the water column in the heated tanks on the last days of the experiment. Previous studies demonstrated that *Scenedesmus* cells remain as single cells at warm temperatures and colonize more frequently at lower temperatures (79). Therefore, the lower concentrations of nutrients in the water column in heated tanks may point to more instances of colonized cells in the reference tanks. In our microscopic images, taken toward the end of the experiment, the proportion of individual cells to colonies in the reference tanks (4.06) was slightly lower than in the heated tanks (5.30).

### Model limitations

As with any modeling study, it is important to highlight the successes as well as the shortcomings of our model predictions, which may improve future application of CFM-Phyto-T. There was variation in model predictions among tanks due to the variable *in-situ* measurements in each tank, which the model resolved. However, some of these *in-situ* parameters may not have been the best input for the model. For example, the irradiance measurements were taken using the reflectance of the water, not the light penetrating the water column. Therefore, the irradiance levels may have been lower than reality, which may affect the allocation predictions to photosynthesis, leading to these high fractions of photosynthetic machinery. CFM-Phyto-T’s predictions in some instances more closely aligned with the reference tanks than heated tanks. This suggests factors outside our modeling framework may have been influential in the treatment tanks. Furthermore, this version of CFM-Phyto-T assumes steady-state conditions, where the uptake of nutrients into the cell is balanced by the concentration of nutrients within, making it so the change in concentration of nutrients over time is negligible or zero. When using this model in ecosystem and forecasting models, a dynamic version of the model should be used in which the change in concentration of cellular nutrients with time is variable. This may improve the mechanisms and feedback of CFM-Phyto-T when working with more stochastic environments.

### Ecosystem implications

As waters warm, freshwater lakes are becoming increasingly vulnerable to thermal stratification of the water column, which limits nutrient availability at depth and increases anoxia. A previous study revealed water column stratification caused sediments to release P into the water column, sparking a large phytoplankton bloom (80). Another study observed high cyanobacteria abundance during an extended period (≥3 weeks) of thermal stratification, attributing it to either internal P loading or a more stable water column (81). Our findings reveal temperature alone may be a significant factor in phytoplankton density, as our mesocosms were well mixed and not replenished with nutrients. We also see an increase in dedication to C storage within phytoplankton cells, which may impact zooplankton populations. Freshwater zooplankton have relatively high P content (11, 82), thus requiring lower N:P ratios in phytoplankton to maintain adequate nutrition. Therefore, warming water combined with a P-limited effect may negatively affect the nutrition of zooplankton in this system, which may ultimately impact the nutrition of higher trophic organisms, such as fish. Previous mesocosm work (83) showed decreased levels of phytoplankton with increasing temperature due to heightened grazing. Here, we did not include zooplankton in the mesocosms, which may contribute to the observed difference. In the future, shallow freshwater lakes may experience increased temperatures, which could increase phytoplankton biomass, but zooplankton grazing could balance this population growth, as seen in previous observations (84). However, the low nutritional quality of phytoplankton may also increase zooplankton’s susceptibility to fish predation even when zooplankton biomass is high (85).

As evident in our experiment, increased temperatures are correlated with increased phytoplankton bloom density. This fact alone is important for managers, as this occurred in a P-limited environment. Globally, there have been many efforts to decrease nutrient loading to freshwater lakes to decrease the chance for eutrophication (86–88). If the atmosphere meets (or exceeds) the 4.5°C increase in temperature, these management efforts may be negated and require new strategies for management in these systems. Further investigation into top-down effects may provide insights on the role of zooplankton in future shallow lakes. This may improve our understanding of ecosystem changes and what actions local managers may take to maintain high water quality and other ecosystem services that benefit local tourism, culture, and economy.

## Data Availability

The data and model code for this study can be found in our Zenodo (which relies on Github) repository (DOI: 10.5281/zenodo.13785971).

## SUPPLEMENTAL MATERIAL

Supplemental Material spectrum.00457-25-s0001.docx Table S1 to S3 and Figure S1 to S11.

ASM does not own the copyrights to Supplemental Material that may be linked to, or accessed through, an article. The authors have granted ASM a non-exclusive, world-wide license to publish the Supplemental Material files. Please contact the corresponding author directly for reuse.
