# Supplementary material for: The effect of temperature on phytoplankton physiology: a mesocosm and modeling study
Source: Microbiol Spectr. 2025 Sep 18;13(10):e00457-25. doi: 10.1128/spectrum.00457-25 (PMC12502652; doi:10.1128/spectrum.00457-25)
Supplement: Supplemental Material — Table S1 to S3 and Figure S1 to S11. [file spectrum.00457-25-s0001.docx]

**The effect of temperature on phytoplankton physiology; a mesocosm and modeling study**

Gabrielle Armin^1^, Gergely Boros^2^, Mariann Kis^3^, Máté Burányi^2,3^, Hajnalka Horváth^3^, Krisztina Krassován^3^, Takako Masuda^4^, Gábor Bernát^3*^, and Keisuke Inomura^1*^

^1^Graduate School of Oceanography, University of Rhode Island, Narragansett, RI, USA

^2^Zooplankton and Ecological Interactions Research Group, HUN-REN Balaton Limnological Research Institute, Tihany, Hungary

^3^Aquatic Botany and Microbial Ecology Research Group, HUN-REN Balaton Limnological Research Institute, Tihany, Hungary

^4^Fisheries Resources Institute, Japan Fisheries Research and Education Agency, Shinhamacho, Shiogama, Miyagi, Japan

*Co-Principal Investigators

Corresponding author: Gabrielle Armin, Graduate School of Oceanography, University of Rhode Island, Horn 206, 218 S Ferry Rd, Narragansett, RI, 02874, USA

**Author e-mails (in order as above**): garmin@uri.edu, boros.gergely@blki.hu, kis.mariann@blki.hu, buranyi.mate@blki.hu, horvath.hajnalka@blki.hu, krassovan.krisztina@blki.hu, takakom@affrc.go.jp, bernat.gabor@blki.hu, and inomura@uri.edu

**Running head:** Modeling algal response to warming in mesocosms

**Keywords:** phytoplankton, temperature, elemental stoichiometry, macromolecular allocation, model, mesocosm, climate change

| 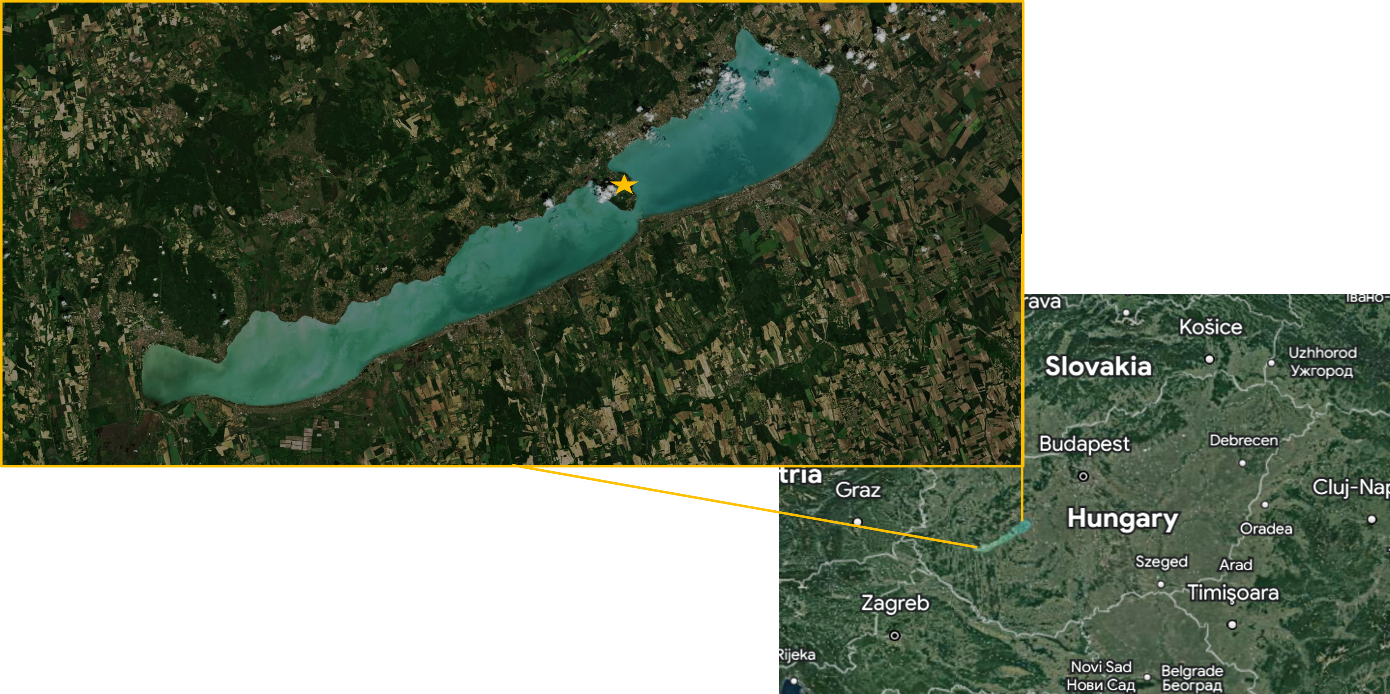 |
| --- |
| Figure S1. Lake Balaton is an oligotrophic, freshwater lake located in western Hungary that is economically and culturally significant to the region. In this study, we conducted a mesocosm experiment at the Balaton Limnological Research Institute located in Tihany, Hungary (starred on closeup map). |

| Table S1. Parameters measured daily, either by tank sensors or in lab by flow cytometry, and intermittently (approximately every third day). |
| --- |
| \| **Parameter** \| **Frequency** \| **Method** \| \| --- \| --- \| --- \| \| Temperature (⁰C) \| Daily \| *In-situ* sensor measurement \| \| Dissolved oxygen (DO) (mg/L) \| Daily \| *In-situ* sensor measurement \| \| Light irradiance (μmol photons m^-2^ s^-1^) \| Daily \| *In-situ* sensor measurement \| \| pH \| Daily \| *In-situ* sensor measurement \| \| Cell density (cells mL^-1^) \| Daily \| Flow cytometry \| \| Total dissolved nitrogen in water column (TDN) \| Intermittent \| Cd-reduction method of Elliott and Porter. After digestion, spectroscopic method \| \| Total phosphorus (TP) in water column \| Intermittent \| Murphy and Riley method after 1 hour of autoclaving in presence of K_2_S_2_O_8_ \| \| Total dissolved phosphorus (TDP) \| Intermittent \| Murphy and Riley method after 1 hour of autoclaving in presence of K_2_S_2_O_8_ \| \| Particulate P \| Intermittent \| Filter in 10 mL of MQ water, digested in autoclave, followed Murphy and Riley protocol \| \| Particulate C, N \| Intermittent \| Elemental analyzer interfaced to Isotope Ratio Mass Spectrometer \| \| Chlorophyll-*a* \| Intermittent \| UV-Vis spectroscopy after methanolic extraction \| \| RNA \| Intermittent \| Direct-zol^TM^ RNA Miniprep kit \| |

| Table S2. Volumes (mL) of sample filtered for biomass samples. |
| --- |
| \| Treatment \| Tank \| Day of experiment \| Volume filtered (mL) \| \| --- \| --- \| --- \| --- \| \| Reference Eukaryotic (RE) \| 1 \| 0 \| 1000 \| \| 4 \| 1000 \| \| 7 \| 1000 \| \| 11 \| 1000 \| \| 14 \| 1000 \| \| 5 \| 0 \| 2000 \| \| 4 \| 1000 \| \| 7 \| 1000 \| \| 11 \| 1000 \| \| 14 \| 1000 \| \| 9 \| 4 \| 1000 \| \| 7 \| 1000 \| \| 11 \| 1000 \| \| 14 \| 1000 \| \| Heated Eukaryotic (HE) \| 2 \| 4 \| 1000 \| \| 7 \| 1000 \| \| 11 \| 500 \| \| 14 \| 500 \| \| 6 \| 4 \| 1000 \| \| 7 \| 1000 \| \| 11 \| 500 \| \| 14 \| 500 \| \| 12 \| 0 \| 1500 \| \| 4 \| 1000 \| \| 7 \| 1000 \| \| 11 \| 1000 \| \| 14 \| 500 \| \| Reference Mixed Population (RM) \| 3 \| 0 \| 1500 \| \| 4 \| 1000 \| \| 7 \| 2000 \| \| 11 \| 1500 \| \| 14 \| 1000 \| \| 8 \| 4 \| 1000 \| \| 7 \| 2000 \| \| 11 \| 1000 \| \| 14 \| 500 \| \| 10 \| 4 \| 1000 \| \| 7 \| 2000 \| \| 11 \| 1000 \| \| 14 \| 500 \| \| Heated Mixed Population (RM) \| 4 \| 4 \| 2000 \| \| 7 \| 1000 \| \| 11 \| 500 \| \| 14 \| 500 \| \| 7 \| 0 \| 1500 \| \| 4 \| 2000 \| \| 7 \| 1000 \| \| 11 \| 500 \| \| 14 \| 500 \| \| 11 \| 0 \| 1500 \| \| 4 \| 2000 \| \| 7 \| 1000 \| \| 11 \| 1000 \| \| 14 \| 800 \| |

| Table S3. Volumes (mL) of sample filtered for chlorophyll*-a* samples. |
| --- |
| \|  \| Tank \| Day of experiment \| Volume filtered (mL) \| \| --- \| --- \| --- \| --- \| \| Reference Eukaryotic (RE) \| 1 \| 0 \| 300 \| \| 4 \| 100 \| \| 7 \| 100 \| \| 11 \| 200 \| \| 14 \| 100 \| \| 5 \| 0 \| 250 \| \| 4 \| 100 \| \| 7 \| 100 \| \| 11 \| 100 \| \| 14 \| 100 \| \| 9 \| 4 \| 100 \| \| 7 \| 100 \| \| 11 \| 100 \| \| 14 \| 100 \| \| Heated Eukaryotic (HE) \| 2 \| 4 \| 100 \| \| 7 \| 100 \| \| 11 \| 60 \| \| 14 \| 80 \| \| 6 \| 4 \| 100 \| \| 7 \| 100 \| \| 11 \| 50 \| \| 14 \| 50 \| \| 12 \| 0 \| 200 \| \| 4 \| 100 \| \| 7 \| 100 \| \| 11 \| 100 \| \| 14 \| 100 \| \| Reference Mixed Population (RM) \| 3 \| 0 \| 200 \| \| 4 \| 200 \| \| 7 \| 200 \| \| 11 \| 100 \| \| 14 \| 100 \| \| 8 \| 4 \| 200 \| \| 7 \| 200 \| \| 11 \| 100 \| \| 14 \| 100 \| \| 10 \| 4 \| 200 \| \| 7 \| 200 \| \| 11 \| 100 \| \| 14 \| 100 \| \| Heated Mixed Population (RM) \| 4 \| 4 \| 200 \| \| 7 \| 100 \| \| 11 \| 100 \| \| 14 \| 100 \| \| 7 \| 0 \| 200 \| \| 4 \| 200 \| \| 7 \| 100 \| \| 11 \| 100 \| \| 14 \| 100 \| \| 11 \| 0 \| 200 \| \| 4 \| 200 \| \| 7 \| 100 \| \| 11 \| 100 \| \| 14 \| 100 \| |

| 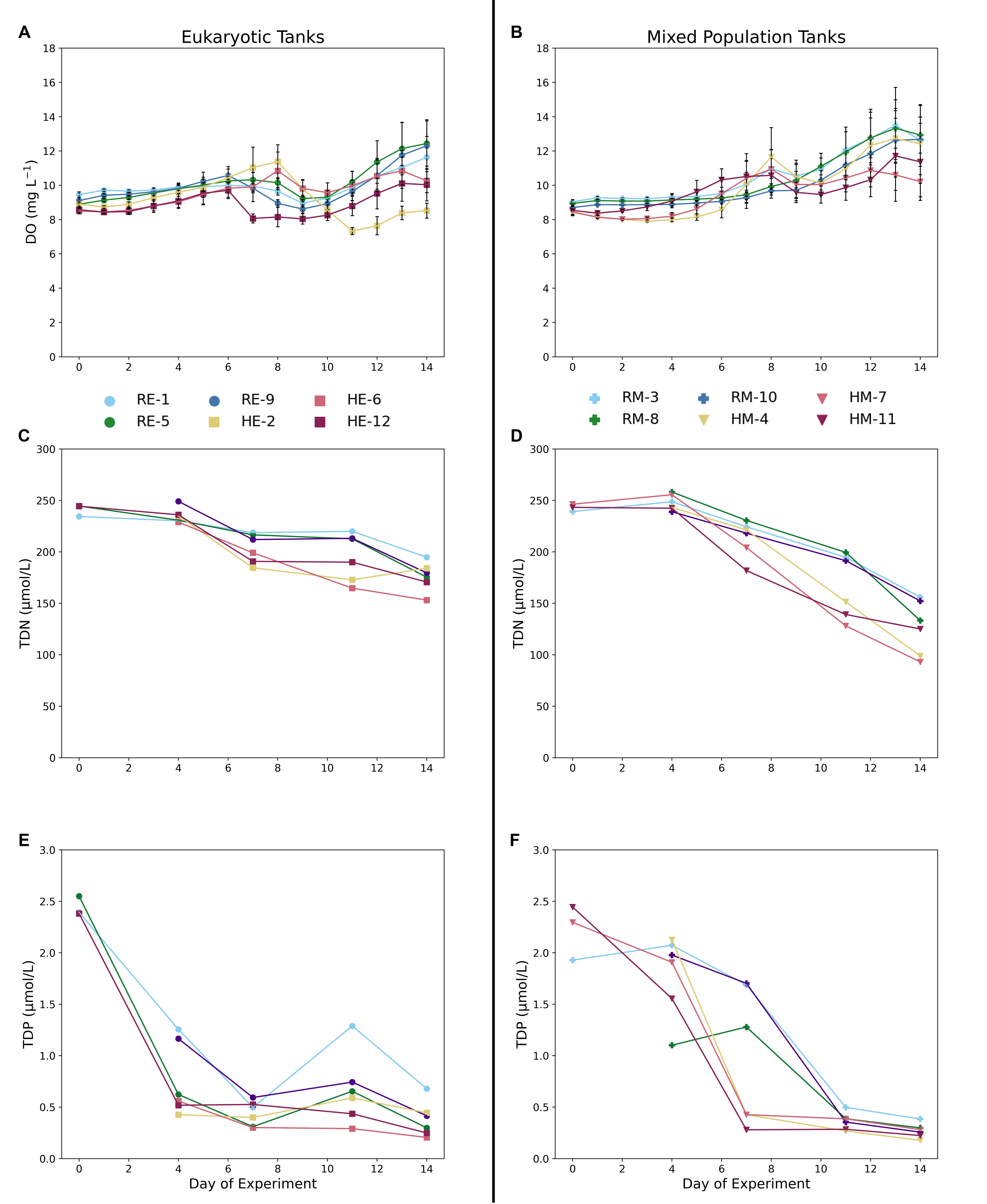 |
| --- |
| Figure S2. Levels of dissolved oxygen (DO, mg L^-1^), total dissolved nitrogen (TDN), μmol N L^-1^), and total dissolved phosphorus (TDP), μmol P L^-1^) in Eukaryotic (left column) and Mixed Population tanks (right column). |

| 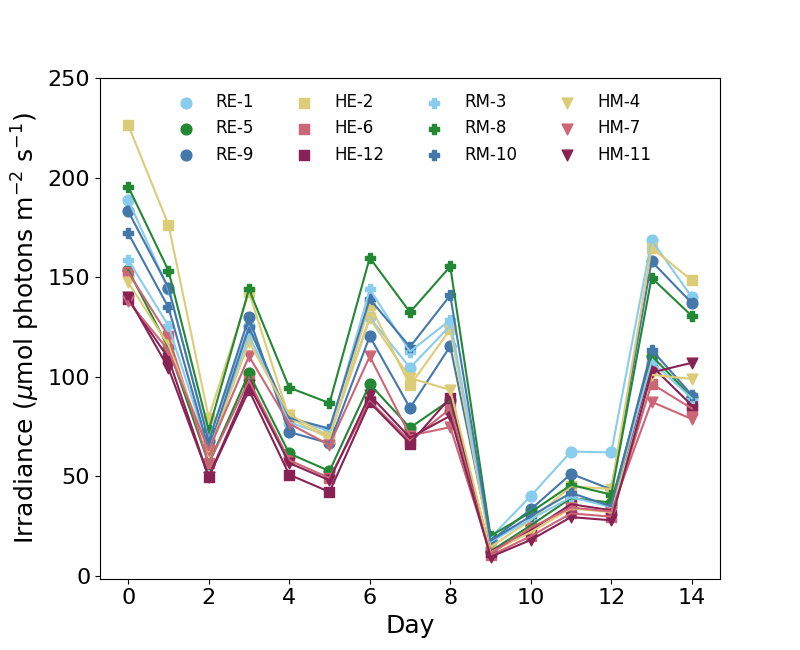 |
| --- |
| Figure S3. Average irradiance levels (μmol photons m^-2^ s^-1^) taken at the time of our grab sampling (7AM-9AM each day) throughout the experiment. |

| 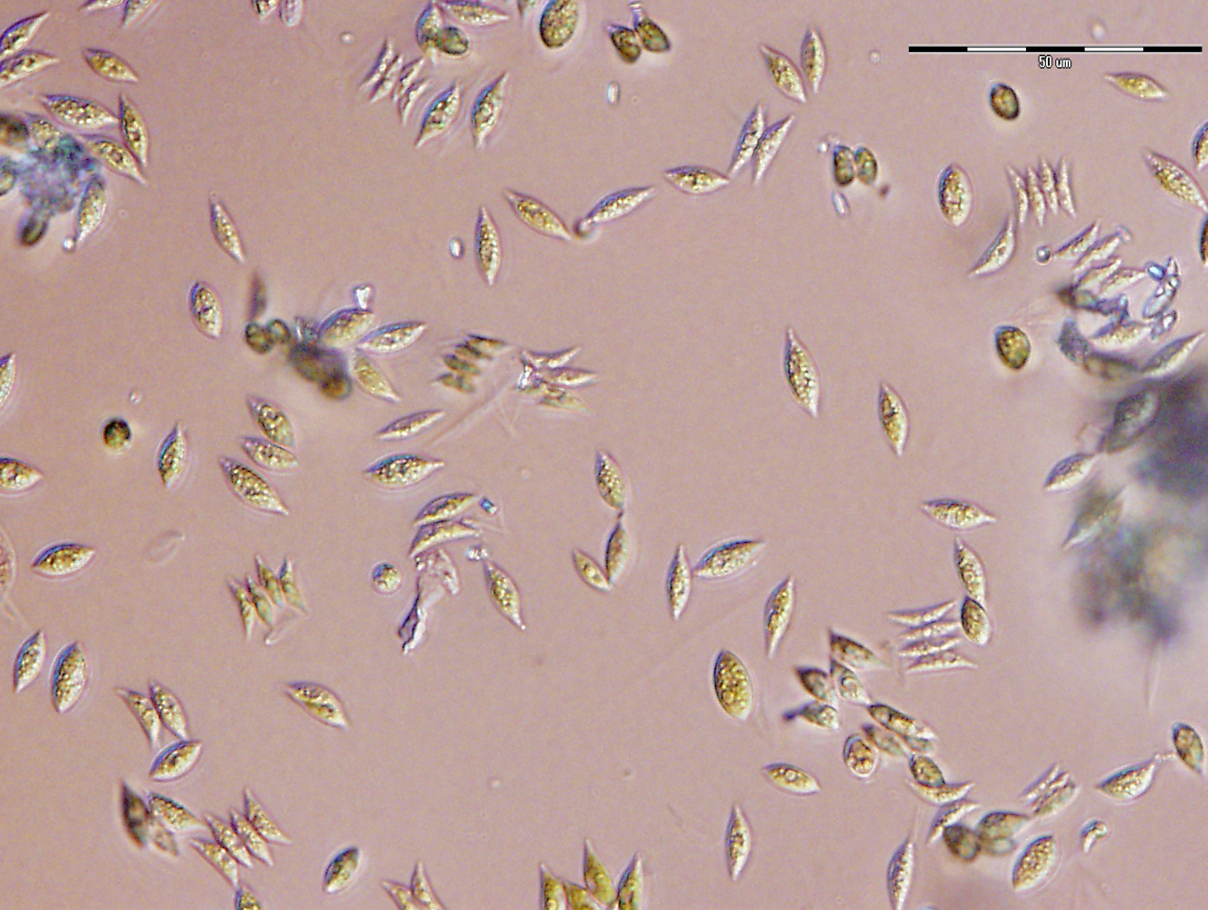 |
| --- |
| Figure S4. Microscope image of a phytoplankton sample from Tank 7 on day 13. Here, we see the colonizing species, *Scenedesmus acutus,* a prevalent species in the Mixed Population tanks and that which began to dominate in the Eukaryotic Tanks on the last day of the experiment. |

| 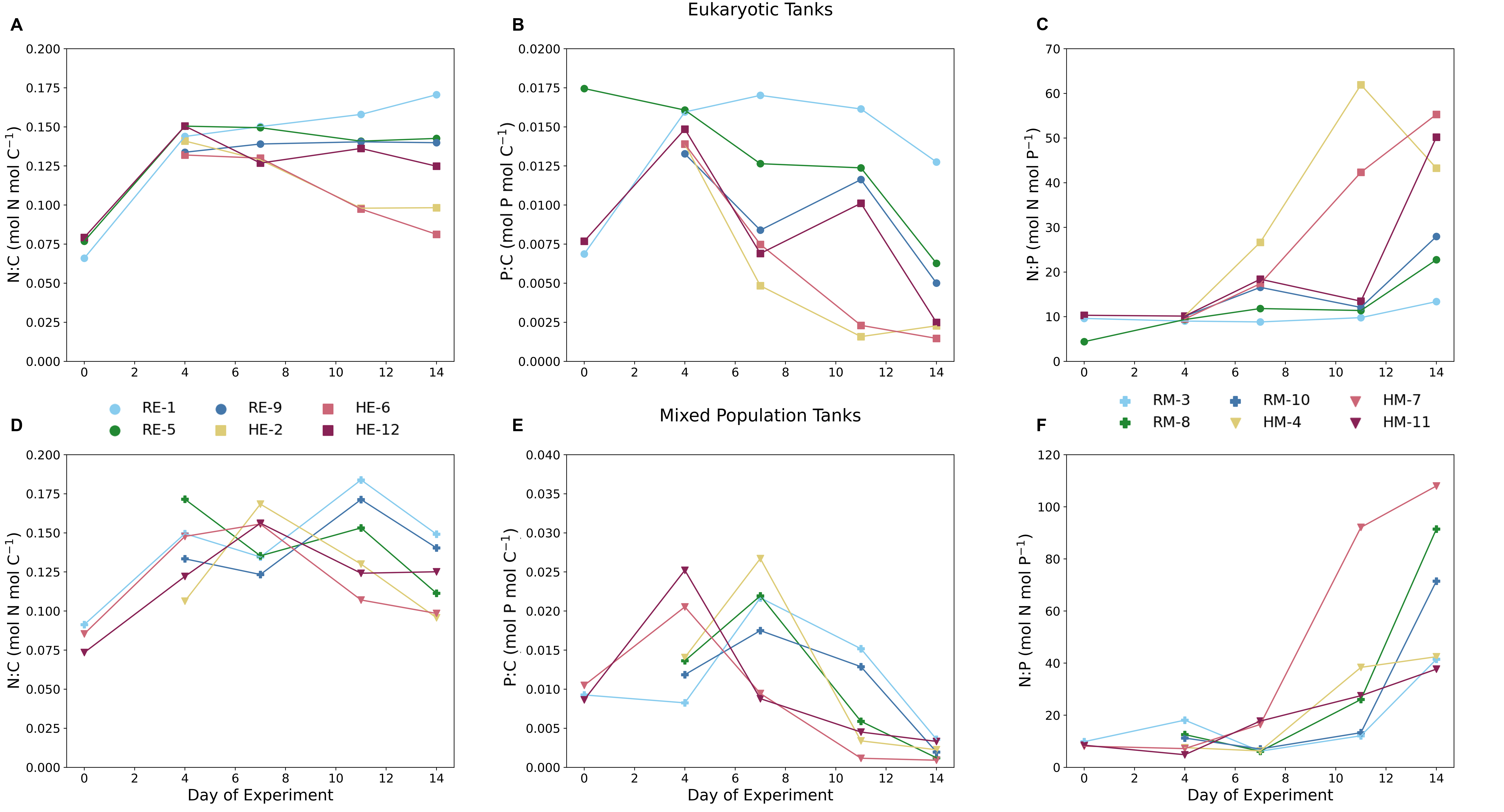 |
| --- |
| Figure S5. Stoichiometry of N:C (mol N mol C^-1^), P:C (mol P mol C^-1^), and N:P (mol N mol P^-1^) in Eukaryotic (A-C) and Mixed Population tanks (D-F). |

| 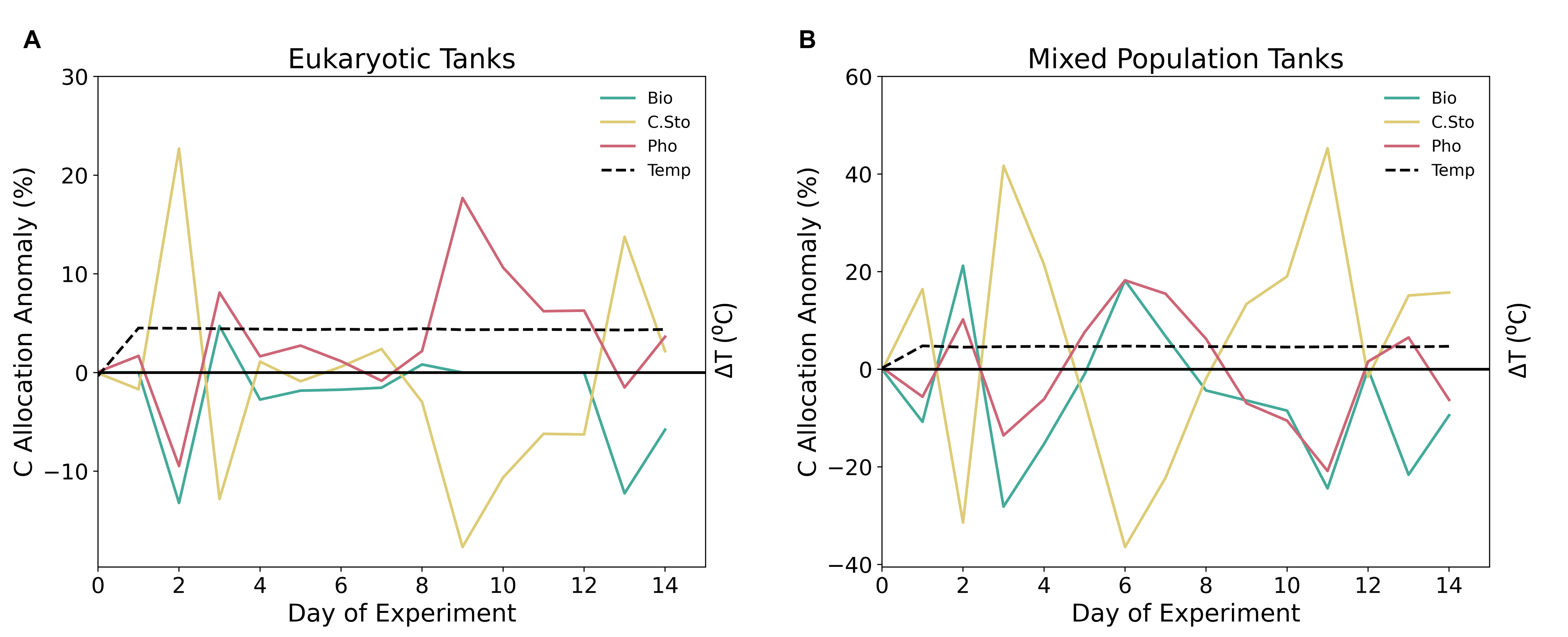 |
| --- |
| Figure S6. Carbon allocation anomalies between heated and reference tanks in Eukaryotic (A) and Mixed Population tanks (B). Anomalies in C allocation for biosynthetic (Bio, teal), photosynthetic (Pho, pink), and carbon storage (C. Sto, yellow) macromolecular pools. Allocation for other pools (i.e., for Nitrogen storage and Essential macromolecules) remained constant, and thus are not shown in the figure. Black, solid line lies at zero to allow for easier visual distinction. Temperature difference (Temp) between heated and reference tanks are emphasized by dotted lines. |

| 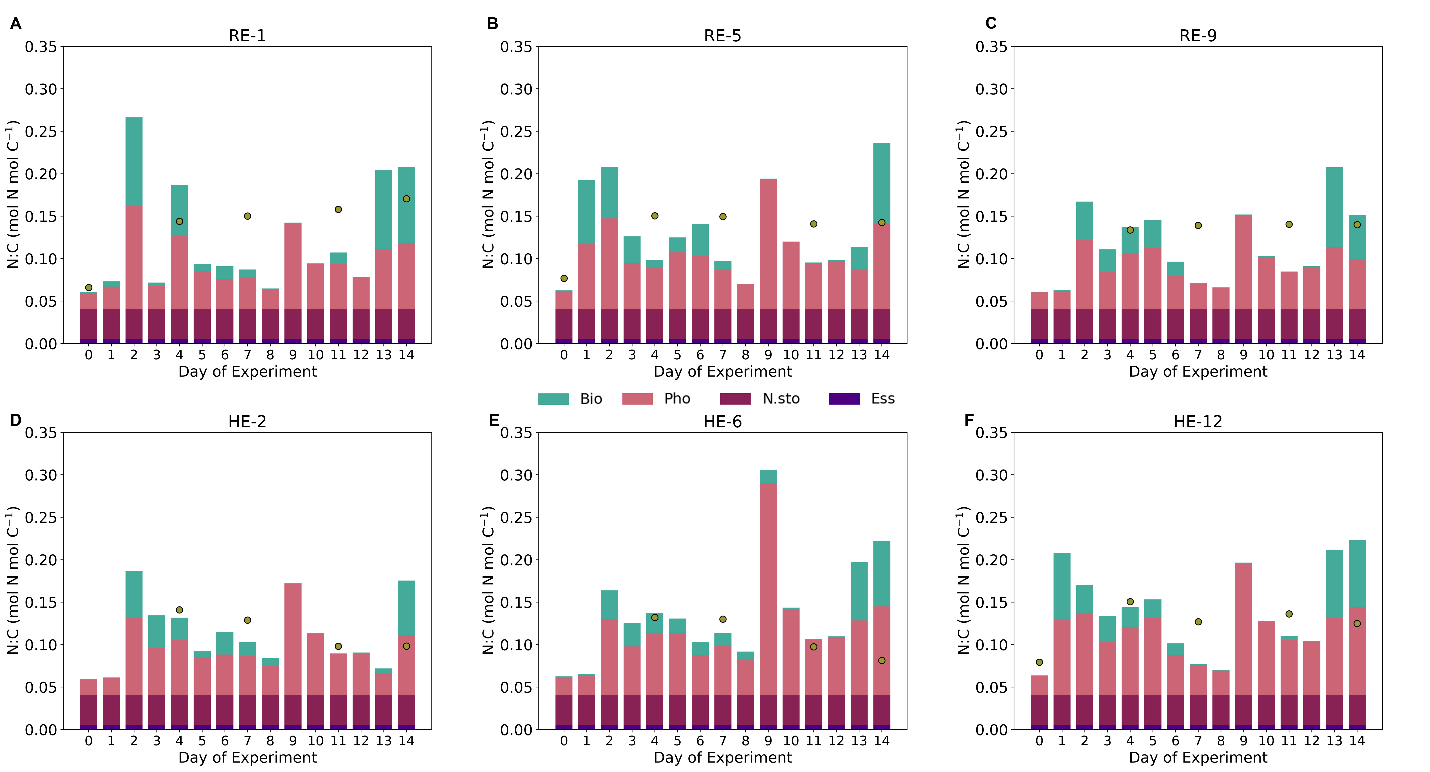 |
| --- |
| Figure S7. Modeled nitrogen allocation (mol N mol C^-1^) to biosynthetic (Bio, teal), photosynthetic (Pho, pink), nitrogen storage (N. Sto, maroon), and essential (Ess, purple) macromolecules for each day of the experiment in the Eukaryotic Tanks. Olive dots represent the experimentally determined N:C values. |

| 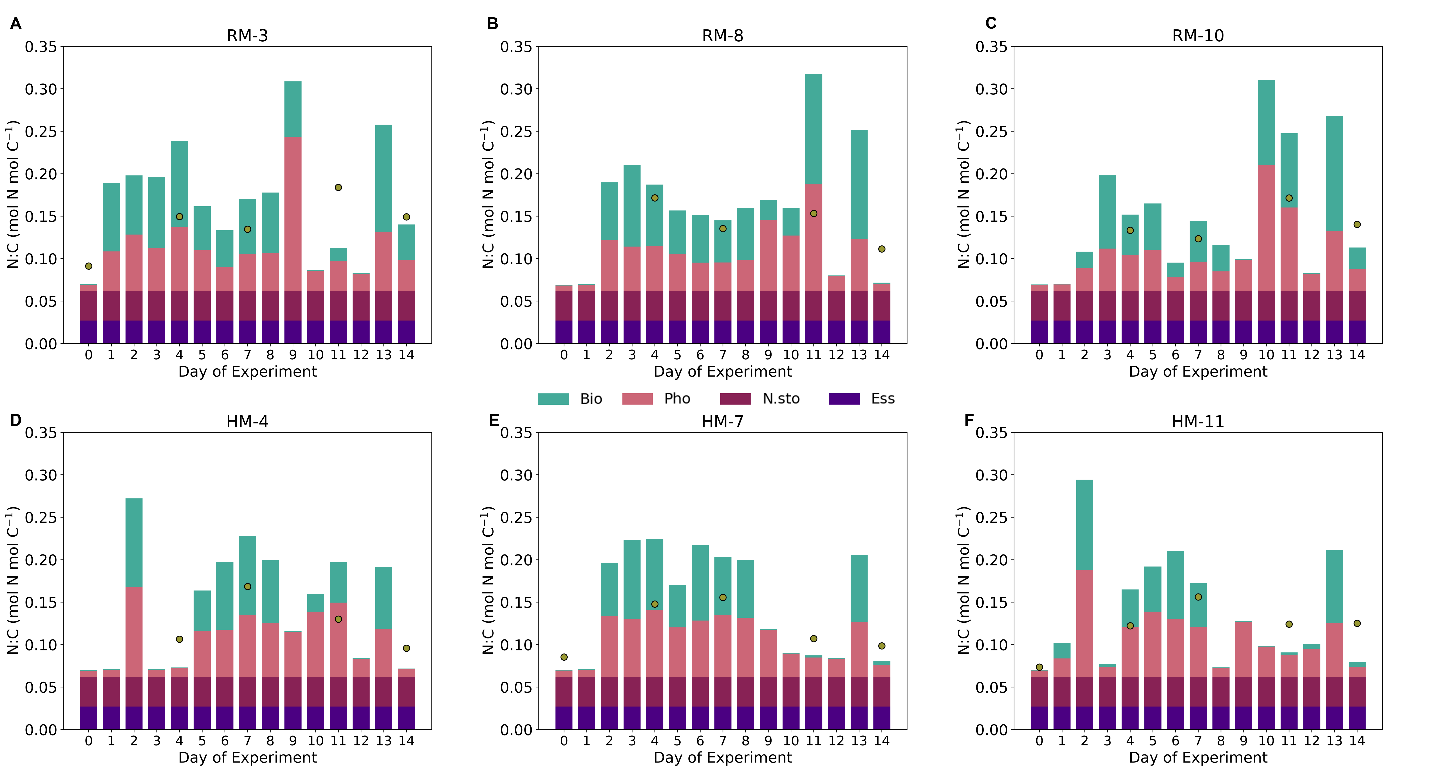 |
| --- |
| Figure S8. Modeled nitrogen allocation (mol N mol C^-1^) to biosynthetic (Bio, teal), photosynthetic (Pho, pink), nitrogen storage (N. Sto, maroon), and essential (Ess, purple) macromolecules for each day of the experiment in the Mixed Population. Olive dots represent the experimentally determined N:C values. |

| 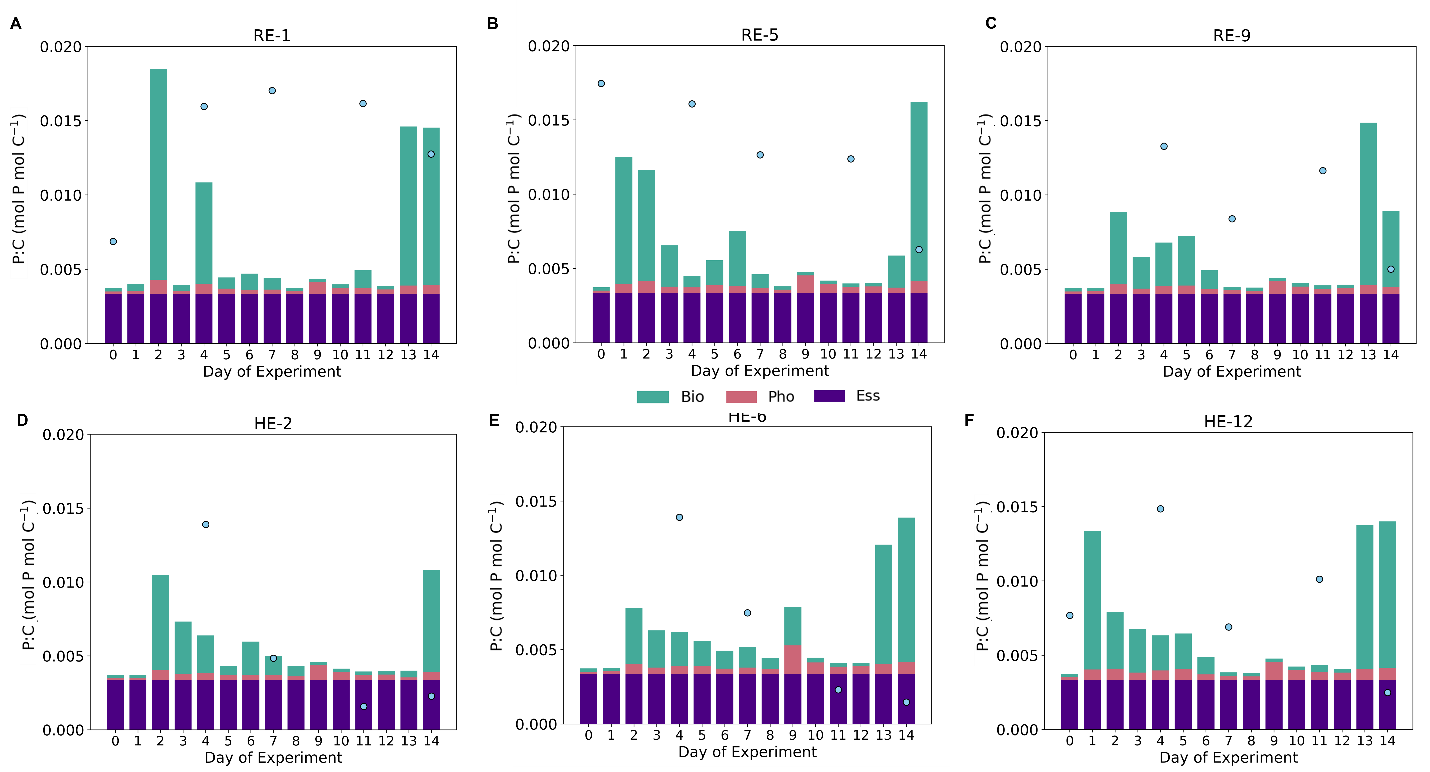 |
| --- |
| Figure S9. Modeled phosphorous allocation (mol P mol C^-1^) to biosynthetic (Bio, teal), photosynthetic (Pho, pink), and essential (Ess, purple) macromolecules for each day of the experiment in the Eukaryotic Tanks. Blue dots represent the experimentally determined P:C values. |

| 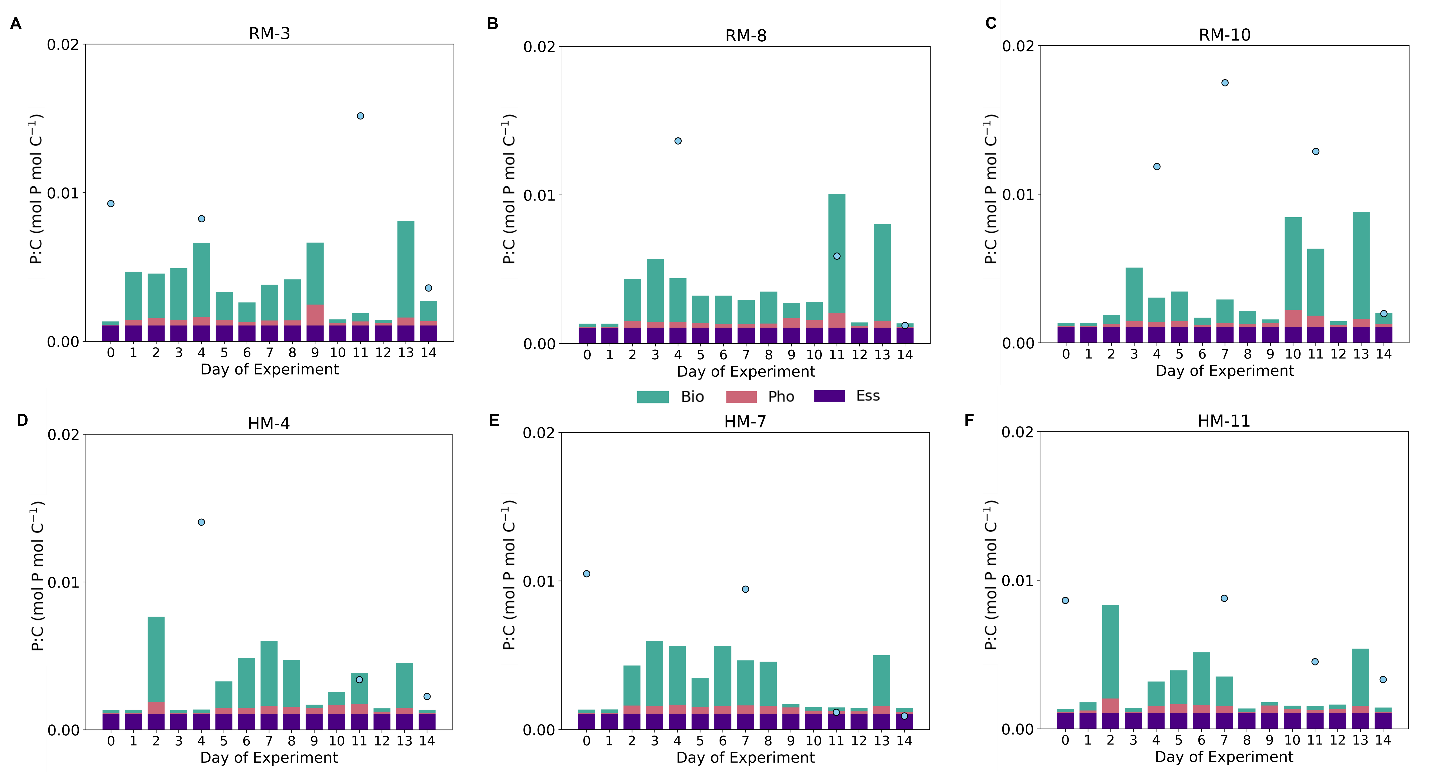 |
| --- |
| Figure S10. Modeled phosphorous allocation (mol P mol C^-1^) to biosynthetic (Bio, teal), photosynthetic (Pho, pink), and essential (Ess, purple) macromolecules for each day of the experiment in the Mixed Population Tanks. Blue dots represent the experimentally determined P:C values. |

| 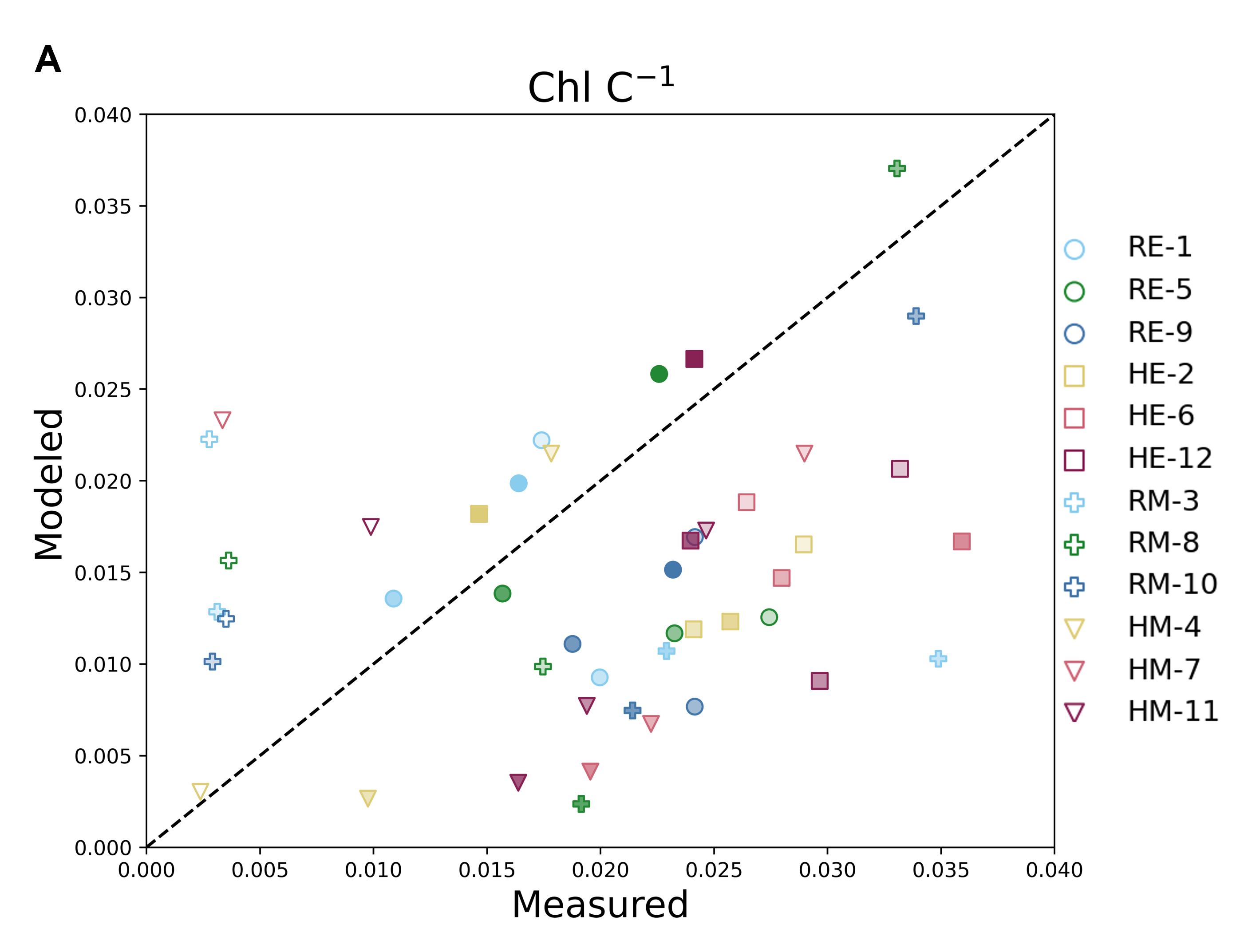 |
| --- |
| Figure S11. Modeled *vs.* Measured values of chlorophyll-*a* (μg Chl μg C^-1^) for all tanks (A). The fill or shading of each point represents the day of the experiment, no fill being the first measurement on day 4 and the opaque points those on day 14. |
